# Osteoimmuno-microbial feedback loops in medication-related osteonecrosis of the jaw: from dysbiosis to persistent structural osteonecrosis

**DOI:** 10.1080/20002297.2026.2751863

**Published:** 2026-10-28

**Authors:** Bingyue Xu, Deqi Hu, Han Lu, Yizhuo Chen, Xin Guo, Keying Liu, Wei He

**Affiliations:** a Department of Oral and Maxillofacial Surgery II, The First Affiliated Hospital of Zhengzhou University, Zhengzhou, Henan, China

**Keywords:** Medication-related osteonecrosis of the jaw, oral microbiome, osteoimmunity, microbial dysbiosis, biofilm, osteonecrosis

## Abstract

**Background:**

Medication-related osteonecrosis of the jaw is a severe adverse event associated with antiresorptive, antiangiogenic and other targeted therapies. Although suppressed bone remodeling and impaired vascularity are central to its pathogenesis, these mechanisms alone do not fully explain the strong clinical associations among local trauma, infection, chronic inflammation and persistent bone exposure.

**Objective:**

To synthesize evidence on microbial dysbiosis, host immune dysregulation, and bone metabolic imbalance in MRONJ, and to propose an integrated osteoimmuno-microbial feedback framework.

**Design:**

This narrative review examined English-language clinical and experimental studies, reviews, and guidelines identified through PubMed, Web of Science, and Scopus, supplemented by reference-list screening. The final search was performed on May 31, 2026.

**Results:**

The reviewed evidence suggests that reduced bone turnover, impaired mucosal repair, and altered immune defense create a vulnerable local niche that permits microbial persistence and multispecies biofilm formation. Dysbiotic biofilms may sustain macrophage-associated inflammatory signaling, disturb bone homeostasis, and contribute to pyroptosis- and necroptosis-related tissue injury. These processes may form a self-perpetuating cycle of microbial colonization, inflammation, bone exposure, and impaired repair. However, microbial dysbiosis has not been established as a universal initiating event.

**Conclusions:**

The proposed framework may help explain progression from potentially reversible local imbalance to persistent structural osteonecrosis. It highlights potential phase-adapted strategies targeting microbial ecology, inflammatory amplification, bone repair, and tissue vitality beyond conventional infection control and debridement.

## Introduction

Medication-related osteonecrosis of the jaw (MRONJ) is an uncommon but severe adverse event associated with antiresorptive agents, including bisphosphonates and denosumab, as well as antiangiogenic and other targeted therapies [1–3]. Clinically, MRONJ is characterized by persistent exposed bone or failure of mucosal healing in the maxillofacial region and is frequently accompanied by pain, infection, functional impairment and reduced quality of life [2–6]. Since the first report of bisphosphonate-associated osteonecrosis of the jaws in 2003, extensive clinical and experimental research has been conducted; however, the mechanisms that determine why MRONJ develops in only a subset of exposed patients remain incompletely understood.

Traditional pathogenic models have emphasized two major processes: suppression of osteoclast-mediated bone remodeling and impairment of local angiogenesis or perfusion [7–13]. These mechanisms are central to the disease, but they do not fully explain several important clinical observations. First, MRONJ occurs only in a minority of patients receiving antiresorptive therapy, even among those with prolonged drug exposure [14–16]. Second, disease onset is often associated with local inflammatory or traumatic triggers, including tooth extraction, periodontitis, peri-apical infection and other dentoalveolar procedures [17–20]. Third, established lesions frequently display clinical, radiographic and histopathological features that overlap with chronic osteomyelitis [21–24]. These observations suggest that drug-induced suppression of bone turnover is necessary but not sufficient to explain the full pathobiology of MRONJ.

Microbial colonization and biofilm persistence have therefore attracted increasing attention. In a drug-altered local environment characterized by reduced bone turnover, delayed mucosal repair and impaired host defense, oral microorganisms may more readily colonize exposed or injured bone surfaces and contribute to chronic inflammation [21–32]. Anaerobic and polymicrobial communities, including Actinomyces-associated organisms and periodontal pathogens, have been reported in MRONJ lesions using histological and molecular approaches [25–32]. However, the pathogenic role of microorganisms remains controversial. It is still unclear whether they mainly represent secondary colonizers of already necrotic bone or whether dysbiotic biofilms can actively amplify inflammation and promote lesion progression in a susceptible jawbone microenvironment.

Recent evidence also indicates that antiresorptive drugs can influence the local immune milieu in addition to their direct effects on bone cells [33–35]. Bisphosphonates may affect macrophage and neutrophil function through mevalonate-pathway inhibition, impaired prenylation and altered innate immune signaling [7–9,36–38]. In this setting, microbial products such as lipopolysaccharide can activate Toll-like receptor 4/NF-κB signaling, promote nucleotide-binding oligomerization domain-like receptor protein 3 inflammasome activation and enhance the release of inflammatory mediators [39–41]. These immune signals may further disturb the balance between receptor activator of nuclear factor-κB ligand and osteoprotegerin, thereby linking microbial stimulation and inflammatory amplification to local bone metabolic imbalance [11,33–35]. At the same time, the oral microbiota should not be viewed simply as a collection of pathogens. Commensal microbial communities contribute to epithelial integrity, immune tolerance and colonization resistance, suggesting that MRONJ may be better understood as a disorder of host–microbe imbalance rather than as a single-pathogen infection [42–46].

A key unresolved question is therefore whether infection in MRONJ is merely a secondary consequence of bone exposure or an active contributor to disease propagation. This distinction has important clinical implications. If microbial colonization is mainly secondary, treatment should focus primarily on restoring bone homeostasis and removing necrotic tissue. If dysbiotic biofilms and inflammatory immune responses actively sustain disease progression, then strategies targeting microbial ecology, immune amplification and tissue repair may be required in addition to conventional antimicrobial therapy and debridement. In this Review, we synthesize clinical and experimental evidence on microbial dysbiosis, biofilm formation, macrophage polarization, inflammasome activation, inflammatory bone remodeling and programmed cell death in MRONJ. We propose an osteoimmuno-microbial feedback model in which a drug-induced susceptible bone niche permits microbial persistence, while dysbiotic biofilms and host inflammatory responses reinforce each other through interlinked pathways involving IL-1β signaling, RANKL/OPG imbalance and pyroptosis- and necroptosis-related tissue injury. This framework is used to organize the evidence for stage-related disease progression and to identify potential intervention points for future mechanism-based prevention and treatment strategies. Based on these observations, we propose a closed-loop osteoimmuno-microbial model in which drug-induced local susceptibility, microbial dysbiosis, biofilm maturation, inflammatory amplification, bone metabolic imbalance and programmed cell death interact to drive persistent osteonecrosis (Figure 1).

**Figure 1.**
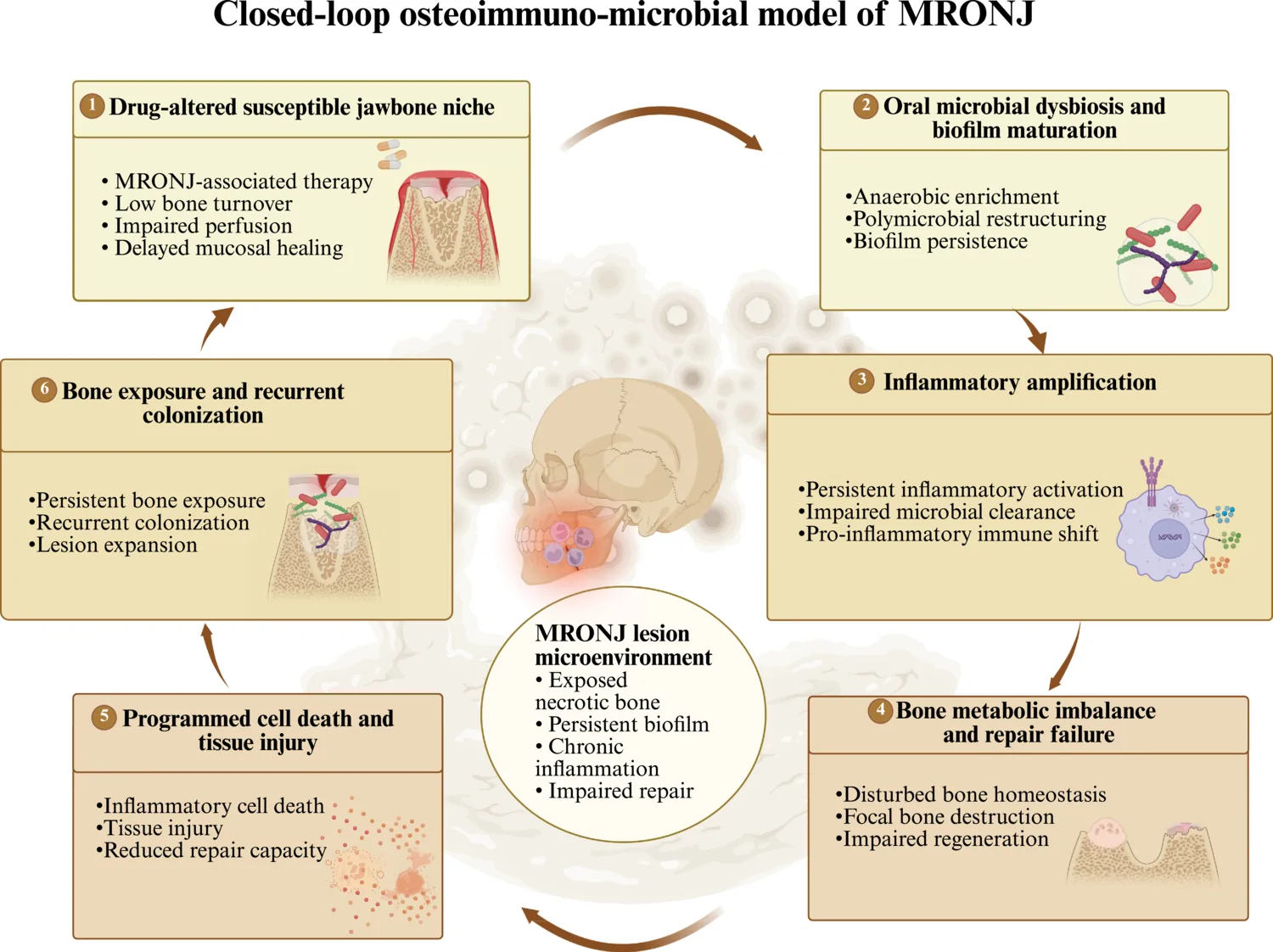
Closed-loop osteoimmuno-microbial model of MRONJ. This conceptual model illustrates the proposed osteoimmuno-microbial feedback loop underlying the initiation and progression of MRONJ. Drug-related alterations in the jawbone microenvironment, including impaired bone turnover, reduced perfusion and delayed mucosal repair, create a susceptible niche that facilitates oral microbial dysbiosis and biofilm maturation. Persistent microbial imbalance may promote sustained immune-inflammatory activation, impaired microbial clearance and progressive disruption of bone homeostasis. Subsequent tissue injury, inflammatory cell death and impaired repair capacity contribute to bone exposure and recurrent microbial colonization, further reinforcing the pathological feedback loop. The model emphasizes the dynamic interaction among drug-altered host susceptibility, microbial communities, immune responses and bone remodeling failure rather than a single pathogenic mechanism.

### Literature search strategy

A narrative literature search was conducted between October 2025 and March 2026 to identify relevant studies on medication-related osteonecrosis of the jaw, oral microbiota, biofilm formation, osteoimmune regulation, macrophage polarization, inflammasome activation, bone metabolic regulation, programmed cell death, multi-omics profiling and computational risk prediction. PubMed, Web of Science and Scopus were searched using combinations of the following terms: ‘medication-related osteonecrosis of the jaw’, ‘MRONJ’, ‘bisphosphonate-related osteonecrosis of the jaw’, ‘oral microbiota’, ‘oral microbiome’, ‘dysbiosis’, ‘biofilm’, ‘Actinomyces’, ‘periodontal pathogens’, ‘macrophage polarization’, ‘NLRP3 inflammasome’, ‘IL-1β’, ‘RANKL’, ‘OPG’, ‘pyroptosis’, ‘necroptosis’, ‘programmed cell death’, ‘proteomics’, ‘metabolomics’, ‘transcriptomics’, ‘multi-omics’, ‘genetics’, ‘pharmacogenomics’, ‘artificial intelligence’, ‘machine learning’, ‘radiomics’ and ‘risk prediction’. The final search was performed on May 31, 2026. Clinical studies, experimental studies, systematic reviews, consensus statements, clinical guidelines and mechanistic review articles published in English were considered. Additional references were identified by manually screening the reference lists of key articles.

Articles were selected according to their relevance to the proposed osteoimmuno-microbial framework, scientific rigor, mechanistic importance and contribution to current understanding of medication-related osteonecrosis of the jaw. Particular attention was given to studies addressing microbial dysbiosis, biofilm persistence, host immune responses, inflammatory bone remodeling, cell-death-related tissue injury, multi-omics characterization and computational prediction. Because this article is intended as a narrative mechanistic review rather than a systematic review or meta-analysis, formal risk-of-bias assessment, quantitative evidence synthesis and protocol registration were not performed. Accordingly, the present article should be regarded as a narrative review.

## Microbial dysbiosis in a drug-altered jawbone niche

The pathogenesis of medication-related osteonecrosis of the jaw (MRONJ) cannot be explained solely by suppression of bone remodeling. Accumulating evidence suggests that long-term antiresorptive therapy can alter the local jawbone microenvironment, interfere with immune regulation and disturb oral microbial homeostasis, thereby contributing to disease initiation and progression [5,6,28–35]. Conventional models centered on osteoclast inhibition and impaired angiogenesis explain important aspects of MRONJ, but they do not fully account for why lesions often become chronic, infected and progressive [7–13,33]. Once microbial colonization and local inflammation are established, the drug-altered niche may sustain both processes and promote a self-reinforcing cycle of infection, inflammation and tissue injury [21–32].

Under physiological conditions, the oral microbiome contains bacteria, fungi, viruses and other microorganisms that coexist in a dynamic ecological equilibrium. Niche competition, metabolic interdependence, epithelial barrier integrity and mucosal immune tolerance help maintain host–microbe homeostasis [42–46]. This balance depends on an intact mucosal barrier, effective immune surveillance and continuous epithelial and bone turnover [7–11,33,42–46]. When mucosal integrity is disrupted or local immune regulation is impaired, the oral ecosystem may shift toward inflammation-associated dysbiosis, allowing conditionally pathogenic species to expand and organize into persistent polymicrobial communities [28–32,42–46].

Continuous antiresorptive therapy may place jawbone in a low-turnover and poorly regenerative state [7–12,33]. Reduced osteoclast activity can limit physiological remodeling and impair the clearance of damaged or necrotic bone [7–11,33]. In parallel, impaired angiogenesis, reduced local oxygen delivery and delayed mucosal repair may further weaken tissue regeneration and wound closure [12,13,33,34]. Bisphosphonates may also affect macrophage and neutrophil function, shifting the local immune milieu toward persistent inflammatory activation and impaired microbial clearance [34–38]. Together, these changes destabilize the interaction among bone tissue, host immunity and the resident oral microbiota, creating a vulnerable microenvironment that favors microbial persistence, biofilm formation and chronic inflammation [21–38,42–46].

### Drug-induced susceptible bone microenvironment

Antiresorptive agents, including bisphosphonates and denosumab, suppress bone remodeling by inhibiting osteoclast differentiation, survival and function or by blocking RANKL-dependent osteoclastogenesis [7–11,33,47]. However, the composition of this susceptible niche is unlikely to be identical across drug classes. Under physiological conditions, continuous bone remodeling repairs microdamage, removes nonviable tissue and preserves skeletal integrity [7–11,33,47]. During sustained antiresorptive exposure, reduced osteoclastic activity and slowed remodeling may impair the clearance of damaged or necrotic bone and compromise local jawbone repair [7–11,33,47]. In parallel, impaired angiogenesis, reduced microvascular support and compromised oxygen delivery may further restrict tissue regeneration [12,13,33,47]. The resulting low-turnover, poorly vascularized and poorly regenerative niche may provide a permissive substrate for microbial persistence after mucosal injury, tooth extraction or bone exposure [17–32,47,48].

Antiresorptive drugs may also disturb mucosal barrier function and local immune defense [34–38,48–51]. Bisphosphonates can impair oral epithelial and fibroblast function, inhibit epithelial migration, delay extraction-socket healing and weaken post-traumatic mucosal repair [48–51]. Such barrier disruption may weaken colonization resistance and facilitate the entry of anaerobic and periodontal-associated microorganisms into extraction wounds, periodontal pockets and exposed or necrotic bone surfaces [17–32,48]. In addition, bisphosphonates may alter neutrophil and macrophage responses, including phagocytic activity, inflammatory mediator production and M1-like polarization [34–38]. Therefore, the drug-altered niche may not only permit microbial colonization but also reduce microbial clearance and prolong inflammatory signaling.

#### Drug-class-specific remodeling of the susceptible jawbone niche

Different MRONJ-associated drug classes may converge on a susceptible jawbone niche through distinct biological entry points. Bisphosphonates accumulate in mineralized bone and suppress osteoclast function through inhibition of the mevalonate pathway and protein prenylation; they may also impair macrophage and neutrophil function and delay mucosal repair. By contrast, denosumab suppresses osteoclastogenesis through RANKL blockade without incorporation into the bone matrix, suggesting a different temporal pattern of remodeling suppression and recovery after drug withdrawal. Antiangiogenic agents primarily compromise vascular and endothelial support, reducing local perfusion, angiogenesis and wound-healing capacity. These differences may influence when and how microbial dysbiosis and immune dysregulation emerge. A predominantly low-turnover or immune-impaired niche may favor prolonged microbial persistence and defective clearance, whereas a poorly perfused and hypoxic niche may impair barrier repair and immune-cell access, thereby facilitating biofilm persistence after local injury. Nevertheless, direct evidence demonstrating drug-specific microbial signatures or immune trajectories in human MRONJ remains limited. Thus, the proposed model should be interpreted as a convergent framework in which different drug classes may alter the relative contribution and timing of bone remodeling, vascular, mucosal and immune disturbances rather than producing an identical pathological niche.

#### Systemic host modifiers of the susceptible niche

Systemic host factors may further modify the drug-altered jawbone niche and influence the strength of the bone–immune–microbiota feedback loop. Diabetes may impair microvascular perfusion, neutrophil and macrophage function, and wound healing, thereby weakening microbial clearance and prolonging local inflammation. Hyperglycemia has also been associated with alterations in the oral microbial ecosystem, although direct evidence linking diabetes-associated dysbiosis to MRONJ progression remains limited. Smoking may further compromise mucosal perfusion and epithelial repair, increase oxidative stress, and alter the oral microbial environment, potentially favoring persistent biofilm formation after local injury. Concomitant corticosteroids or other immunosuppressive therapies may reduce antimicrobial immune responses and tissue-repair capacity, thereby facilitating microbial persistence and delaying inflammatory resolution. Collectively, these systemic factors may act as host modifiers that lower the threshold at which a drug-altered jawbone niche progresses toward self-sustaining microbial persistence, immune dysregulation and structural tissue injury. However, direct longitudinal evidence demonstrating that these factors produce specific microbiome trajectories in MRONJ is still limited.

### Microbial colonization and multispecies network restructuring

Anaerobic and polymicrobial communities have been repeatedly identified in MRONJ lesions using histopathological, culture-based and molecular approaches [21–32,52]. Actinomyces-associated colonies are frequently reported in necrotic jaw specimens, and targeted histological evaluation has further highlighted the high frequency of Actinomyces infection in MRONJ tissues [25–27]. However, sequencing-based studies suggest that MRONJ is unlikely to be explained by a single causative microorganism. Instead, microbial changes often involve multiple organisms associated with periodontitis, gingival disease and endodontic infection, supporting a multifactorial and community-based model [28–32,52].

Within a drug-induced susceptible niche, normally low-abundance pathobionts may gain ecological advantages [44,53]. Periodontal pathogens and bridging taxa, including Fusobacterium nucleatum, Porphyromonas gingivalis and related anaerobes, may contribute to microbial restructuring through coaggregation, tissue-damaging virulence factors and immune modulation [30,44,53–56]. Fusobacterium nucleatum may act as a bridging organism within oral biofilms, facilitating bacterial coaggregation and community maturation [54,55]. Porphyromonas gingivalis may further contribute to dysbiosis through virulence factors, immune subversion and modulation of host inflammatory responses [53,56]. Experimental evidence also suggests that the relationship between bacteria and MRONJ is not simply pathogenic; commensal microbiota may help maintain ecological balance and protect against inflammation-associated osteonecrosis under specific conditions [46]. These findings support the concept that MRONJ-associated infection should be understood as host–microbe imbalance in a drug-altered jawbone niche rather than as a single-pathogen disease. Although several taxa have been repeatedly reported in MRONJ lesions, no MRONJ-specific core pathogen or reproducible pathogenic consortium has yet been established. Actinomyces-associated organisms are among the most consistently detected microorganisms in histopathological studies of necrotic jaw specimens, suggesting a close association with established lesions. However, their frequent detection in exposed necrotic bone does not by itself demonstrate a primary causal role. Sequencing-based studies have also reported enrichment of periodontal-associated anaerobes, including Fusobacterium nucleatum and Porphyromonas gingivalis, which may contribute to biofilm maturation, microbial coaggregation and immune modulation. Nevertheless, these organisms are not specific to MRONJ and are also commonly associated with periodontal and other oral inflammatory diseases. Therefore, current evidence favors a community-level dysbiosis model rather than a single-pathogen model. Whether a reproducible MRONJ-associated microbial consortium exists remains unresolved and requires validation across longitudinal, multicenter cohorts using standardized sampling and sequencing methods.

#### Consistency and heterogeneity across sequencing-based microbiome studies

Sequencing-based microbiome studies broadly support microbial community restructuring in MRONJ, although the specific taxonomic findings are not fully consistent. Studies using 16S rRNA sequencing and other microbiological approaches have reported enrichment of anaerobic and periodontal-associated organisms, but the dominant taxa vary across cohorts, supporting a community-level dysbiosis rather than a single-pathogen model [28–32,52]. More recently, shotgun metagenomic analysis identified increased Actinomyces abundance and a set of MRONJ-associated genes encoded by Actinomyces genomic regions, providing preliminary functional evidence beyond taxonomic profiling [57]. However, these findings were derived from a relatively small cohort and remain to be independently validated. Overall, current studies are more consistent in demonstrating ecological dysbiosis and polymicrobial restructuring than in identifying a reproducible disease-specific taxonomic or functional signature. Differences in sampling site, disease stage, medication exposure, prior antibiotic use and sequencing methodology may contribute to the observed heterogeneity.

### Biofilm formation and mechanisms of chronicity

Biofilm formation may help explain why infection associated with MRONJ is persistent, difficult to eradicate and prone to recurrence. Exposed necrotic bone provides an adhesive mineralized surface, while reduced bone turnover, impaired perfusion and delayed mucosal repair limit normal tissue renewal and immune access [12,13,21–25,33,47–51]. In this environment, Actinomyces-associated organisms, periodontal pathobionts and other anaerobes may attach to the bone matrix, aggregate and develop into spatially organized polymicrobial biofilms [24–27,30–32,58,59].

Mature biofilms differ substantially from planktonic bacterial populations [58,59]. Their extracellular polymeric matrix can limit antimicrobial penetration, protect deeper microorganisms from host immune attack and support phenotypic heterogeneity within the microbial community [58,59]. Bacteria in deeper biofilm layers may exhibit reduced metabolic activity, stress-adapted phenotypes and increased tolerance to antimicrobial therapy [58,59]. These properties may contribute to persistent microbial stimulation despite systemic antibiotic treatment and local wound care [32,58,59]. Biofilm communities may also sustain chronic inflammation by continuously releasing microbial products, extracellular DNA and other inflammatory triggers that activate innate immune pathways [39–41,58,59].

The interaction between biofilms and the drug-altered jawbone niche is therefore reciprocal. Necrotic bone facilitates microbial attachment, while biofilms protect microorganisms from antimicrobial and immune clearance [24,25,58,59]. Persistent microbial stimulation can amplify inflammation, further impair tissue repair and expose additional bone surfaces, which in turn provide new substrates for colonization [21–32,47–52,58,59]. This reciprocal relationship may create a local barrier to resolution and help drive the transition from an acute post-traumatic wound to chronic, persistent osteonecrosis.

### The microbe–inflammation–bone destruction amplification loop

Chronic polymicrobial biofilms may continuously release microbial products and inflammatory stimuli that sustain local inflammation in MRONJ lesions [39–41,58,59]. Lipopolysaccharide, lipoproteins, extracellular DNA and other biofilm-derived components can activate innate immune pathways, including Toll-like receptor/NF-κB signaling and inflammasome-related responses [39–41,58,59]. These signals may promote the production of inflammatory mediators such as TNF-*α*, IL-1β, IL-6 and IL-8, recruit neutrophils and macrophages, and favor a persistent pro-inflammatory immune pattern, including M1-like macrophage polarization [33–41,52].

The chronic inflammatory milieu can also disturb bone metabolic regulation. TNF-*α* and IL-1β may increase RANKL expression, reduce OPG-mediated buffering and shift the RANKL/OPG balance toward osteoclastogenic signaling [11,33–35]. This point is important because MRONJ develops in the context of antiresorptive therapy, in which systemic bone turnover is suppressed [7–11,33]. The pathological process is therefore unlikely to reflect generalized bone resorption. Rather, it may involve a local mismatch in which systemic remodeling remains inhibited, while focal inflammatory signaling promotes localized osteoclast-related activity, bone injury and impaired repair [7–11,33–35,52]. Clinical and molecular studies of MRONJ lesions support a relationship among microbial dysbiosis, inflammatory immune activation and altered bone homeostasis [28–32,52].

As inflammation persists, necrotic bone may expand and exposed mineralized surfaces may increase, creating additional substrates for bacterial attachment and biofilm maturation [21–27,58,59]. In a low-turnover and poorly vascularized jawbone niche, clearance of necrotic bone is reduced, inflammatory resolution is delayed and regenerative capacity is weakened [12,13,33,47–51]. These processes may initiate a self-reinforcing cycle of microbial persistence, inflammatory amplification, bone injury, bone exposure and recurrent colonization [21–32,47–55,58–60]. Microorganisms in MRONJ should therefore not be regarded only as passive contaminants of exposed tissue. In susceptible lesions, they may also act as stage-dependent amplifiers of chronic inflammation and local bone damage [21–32,52,58,59].

### The dual role of infection: initiator or amplifier?

Although increasing evidence supports the contribution of infection and microbial dysbiosis to MRONJ, the exact role of microorganisms remains unresolved. One interpretation is that infection occurs mainly after osteonecrosis has developed. From this perspective, drug-induced suppression of bone remodeling, impaired vascularity and delayed mucosal repair create the primary pathological substrate, whereas oral bacteria secondarily colonize exposed necrotic bone and aggravate local inflammation [2–13,21–24,47]. This view is supported by the fact that many microbiome studies analyze established lesions, making it difficult to distinguish early microbial drivers from secondary colonization.

Other evidence suggests that microorganisms may participate earlier in disease development under specific conditions [28–32,52]. Local infectious or inflammatory triggers, including tooth extraction sites, periodontitis, peri-apical disease and periodontal pathogens, are associated with increased MRONJ risk or lesion development in clinical and experimental settings [17–32,46,52]. Once bacteria enter a drug-altered niche, they may not simply occupy necrotic tissue passively. Instead, dysbiotic communities and biofilms may amplify innate immune activation, disturb bone metabolic signaling and impair tissue repair [33–41,52–56,58,59].

Animal studies further highlight the complexity of microbial participation. Experimental evidence indicates that commensal microbiota may protect against MRONJ development in a periapical disease model, suggesting that not all microorganisms should be considered pathogenic in this context [46]. Therefore, microbial involvement in MRONJ is likely to depend on host immunity, medication exposure, microbial community structure, mucosal barrier status, local tissue perfusion and disease stage. This supports a host–microbe imbalance model rather than a simple infection model.

Taken together, current evidence does not establish microbial dysbiosis as a universal initiating event in MRONJ. A substantial proportion of clinical microbiome and histopathological studies have examined established lesions after bone exposure or necrosis, and therefore cannot exclude the possibility that microbial colonization is secondary to tissue breakdown. At the same time, associations between local dental infection, periodontal or periapical inflammation and MRONJ development, together with experimental evidence, suggest that microorganisms may contribute earlier under susceptible conditions. We therefore interpret dysbiotic biofilms primarily as context- and stage-dependent amplifiers rather than obligatory initiating drivers. In this framework, drug-induced impairment of bone turnover, vascular support and mucosal repair may create the initial susceptible niche, whereas microbial dysbiosis may subsequently sustain inflammation, impair resolution and accelerate structural progression.

From a disease-progression perspective, infection should be viewed as a dynamic and stage-specific process rather than simply as either an initiating event or a secondary complication. In early phases, medication-induced suppression of bone turnover, mucosal barrier injury and altered immune defense may permit microbial colonization [7–13,33–38,47–51]. In intermediate phases, biofilm maturation and inflammatory signaling may reinforce macrophage activation, inflammasome-related responses and RANKL/OPG imbalance [11,33–41,52–56,58,59]. In later phases, chronic biofilm persistence may maintain inflammation, impair repair and promote further bone exposure [21–32,47–51,58,59]. Thus, microorganisms are best understood as context-dependent contributors to MRONJ progression, with roles that may shift from colonizers to inflammatory amplifiers as the lesion evolves. Taken together, these findings support a model in which a drug-altered jawbone niche permits microbial restructuring and biofilm maturation, thereby sustaining chronic colonization, inflammation and delayed resolution (Figure 2).

**Figure 2.**
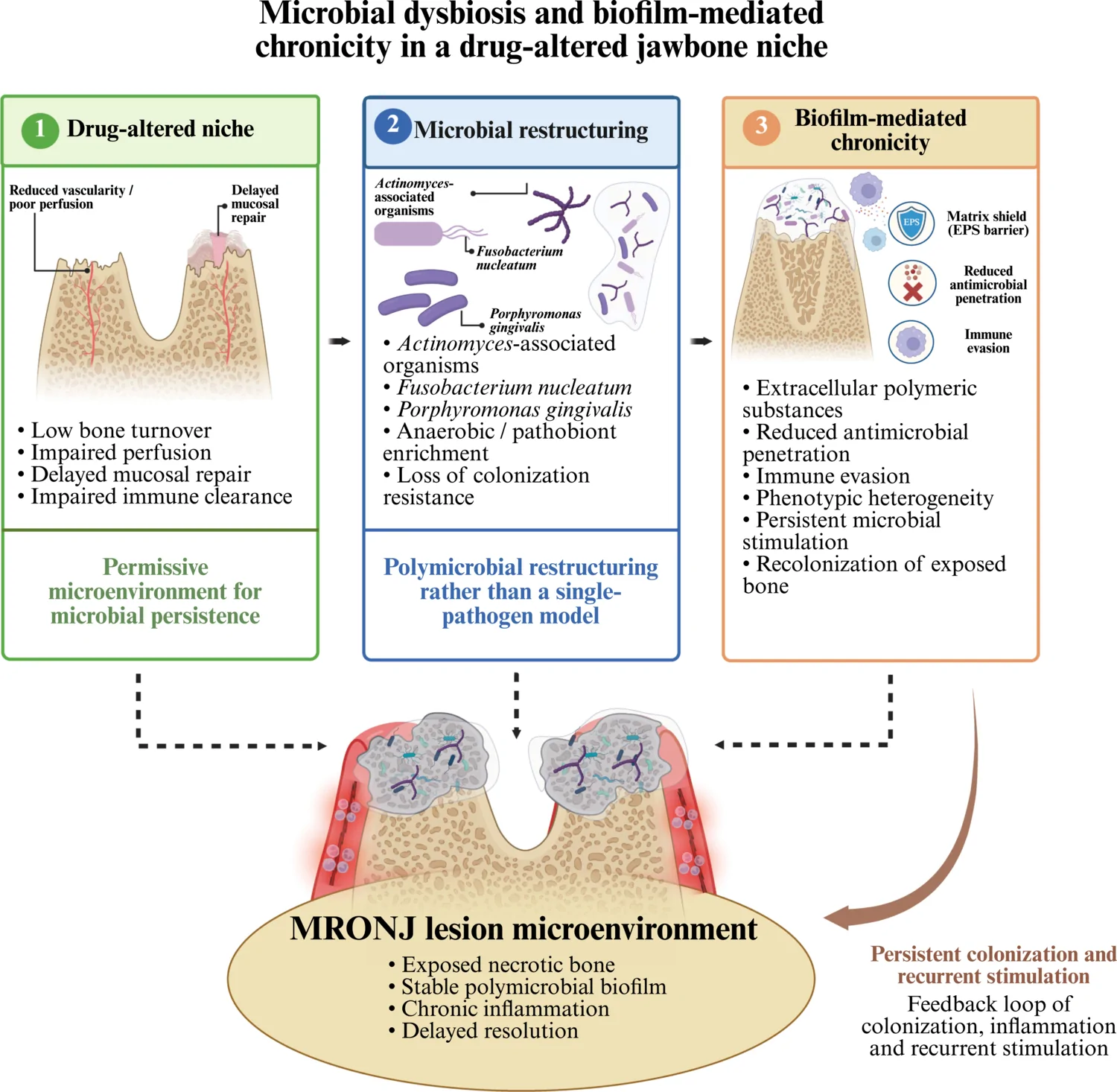
Microbial dysbiosis and biofilm-mediated chronicity in a drug-altered jawbone niche. A drug-altered jawbone niche characterized by low bone turnover, impaired perfusion, delayed mucosal repair and reduced immune clearance may permit microbial persistence after local injury. Within this permissive environment, oral microbial communities may undergo polymicrobial restructuring, including enrichment of Actinomyces-associated organisms, Fusobacterium nucleatum, Porphyromonas gingivalis and other anaerobic or periodontal-associated pathobionts. These changes favor biofilm establishment and maturation on exposed or injured bone surfaces. Mature biofilms can limit antimicrobial penetration, promote immune evasion, support phenotypic heterogeneity and provide persistent microbial stimulation. Together, microbial dysbiosis and biofilm persistence may sustain chronic colonization, inflammation, delayed resolution and recurrent stimulation of exposed bone. MRONJ, medication-related osteonecrosis of the jaw; EPS, extracellular polymeric substances.

## Immune dysregulation and imbalance of bone metabolism

### Imbalanced macrophage polarization

#### Effects of antiresorptive agents on macrophage function

Antiresorptive drugs do not act exclusively on osteoclasts. They may also reshape the local immune microenvironment by altering monocyte and macrophage viability, differentiation and function [34–38,61]. Nitrogen-containing bisphosphonates inhibit the mevalonate pathway and reduce the synthesis of farnesyl pyrophosphate and geranylgeranyl pyrophosphate, thereby impairing the prenylation of small GTPases such as Rho, Rac and Rab [7–9,36,61]. These molecules are important for cytoskeletal remodeling, migration, phagocytosis, vesicular trafficking and lysosomal maturation. Impaired prenylation may therefore compromise phagosome–lysosome fusion and reduce macrophage-mediated microbial clearance [36,61].

Bisphosphonate exposure may also increase macrophage responsiveness to microbial stimuli. In the presence of lipopolysaccharide and other biofilm-derived products, Toll-like receptor 4 activation can promote MyD88-dependent NF-κB and MAPK signaling, leading to the production of inflammatory mediators such as TNF-*α*, IL-1β and IL-6 [38,39,53,58,59,61]. These signals may further converge on inflammasome-related pathways and increase IL-1β maturation, particularly in a microenvironment characterized by persistent microbial stimulation and oxidative stress [40,41,61]. Thus, antiresorptive drugs and microbial signals may interact to generate a local immune state in which inflammatory activation is sustained but antimicrobial clearance remains inefficient.

Under physiological conditions, macrophages can shift dynamically between pro-inflammatory and reparative phenotypes. M1-like macrophages support antimicrobial defense by producing inflammatory mediators, whereas M2-like macrophages contribute to tissue repair, angiogenesis and inflammatory resolution [61,62]. In MRONJ-related environments, this balance may be disrupted. Impaired phagocytosis may reduce pathogen clearance, while persistent biofilm stimulation and drug-induced metabolic stress may favor M1-like polarization and delay the transition toward reparative M2-like responses [34–38,61–63].

#### Persistence of M1-like macrophages and maintenance of chronic inflammation

Persistent microbial stimulation and mature biofilms may provide continuous inflammatory input in MRONJ lesions [39–41,58,59]. Biofilm-derived lipopolysaccharide, lipoproteins, extracellular DNA and other microbial products can repeatedly activate Toll-like receptor/NF-κB signaling and inflammasome-associated responses [39–41,58,59]. These cues may maintain macrophages in a pro-inflammatory state and sustain local production of TNF-*α*, IL-1β, IL-6 and other inflammatory mediators [34,35,38–41,52,61,62].

Clinical and experimental evidence supports the relevance of macrophage polarization in MRONJ. Human MRONJ tissues have shown altered M1/M2 macrophage polarization associated with disease progression [62]. In a mouse model of MRONJ development, M1 macrophage predominance was observed during early lesion formation, and M1-like macrophages were associated with matrix metalloproteinase-13 expression and disruption of the surrounding collagen network. Pharmacological promotion of an M2-like phenotype reduced bone exposure and osteonecrosis, suggesting that an unresolved M1-dominant response may contribute to disease progression [63]. These findings support the concept that macrophage polarization is not merely a bystander phenomenon but may influence whether local inflammation resolves or progresses toward persistent tissue injury.

The persistence of M1-like macrophages may also connect immune dysregulation with bone metabolic imbalance. Pro-inflammatory cytokines such as TNF-*α* and IL-1β can increase RANKL-associated signaling, reduce OPG-mediated buffering and shift the local microenvironment toward osteoclastogenic and matrix-degrading activity [11,34,35,64]. In the setting of systemic antiresorptive therapy, this does not necessarily indicate generalized bone resorption [7–11,33,47]. Rather, it may reflect a focal pathological mismatch in which normal remodeling is suppressed, while local inflammatory signals continue to promote bone injury, matrix degradation and impaired repair [7–11,33–35,47,52,61–64]. In parallel, reactive oxygen species, nitric oxide and matrix-degrading enzymes generated in chronically inflamed tissues may further damage the bone matrix, vascular endothelium and adjacent soft tissues [34,35,61–63].

Resolution of inflammation normally requires a transition from a pro-inflammatory phase to a repair-oriented phase [61–63]. M2-like macrophages produce anti-inflammatory and pro-reparative mediators, support angiogenesis and contribute to tissue remodeling [61–63]. In MRONJ, poor perfusion, low bone turnover, persistent microbial stimulation and prolonged drug exposure may prevent this transition [12,13,33,47–51,58,59,61–63]. As a result, pro-resolution signals may be insufficient, allowing an initially protective antimicrobial response to become chronic, destructive inflammation.

#### Bidirectional crosstalk between macrophages and biofilms

The interaction between macrophages and biofilms is likely bidirectional. Mature biofilms continuously release microbial products that activate Toll-like receptor signaling, inflammasome-related pathways and pro-inflammatory macrophage responses [39–41,58,59]. This persistent stimulation may favor an M1-like phenotype and maintain high levels of inflammatory mediators [61–63]. Conversely, macrophage-derived TNF-*α*, IL-1β, reactive oxygen species and matrix-degrading enzymes may injure local tissues, expose additional mineralized surfaces and create new substrates for bacterial attachment and biofilm maturation [34,35,52,58,59,61–64].

Biofilms can also reduce the effectiveness of macrophage-mediated clearance [58,59]. Their extracellular polymeric matrix restricts immune-cell access to deeper bacterial populations and supports microbial phenotypic heterogeneity [58,59]. As a result, macrophages may remain highly activated but inefficient at eliminating biofilm-protected microorganisms [36,58,59,61]. Some oral pathobionts may further interfere with phagosome maturation, lysosomal function or oxidative killing, although the extent to which these mechanisms operate in human MRONJ lesions requires further validation [36,53,61].

This combination of persistent activation and ineffective clearance may be central to chronic lesion maintenance. Biofilms sustain inflammatory macrophage activation, while macrophage-derived injury expands the ecological niche available for biofilm growth. The result is a self-reinforcing macrophage–biofilm axis that links microbial persistence, inflammatory amplification and progressive tissue damage in MRONJ.

Although macrophages are emphasized in the present model, they are unlikely to act in isolation. Other innate and adaptive immune cells may also participate in the bone–immune–microbiota feedback loop. Neutrophils represent an important first-line defense against oral microorganisms, and bisphosphonate exposure has been reported to impair their chemotaxis, phagocytosis and oxidative burst, potentially reducing microbial clearance and prolonging inflammatory stimulation. T cells may further connect persistent microbial inflammation with bone remodeling through cytokine production and RANKL expression, thereby contributing to osteoimmune imbalance. Dendritic cells are also biologically positioned to link microbial sensing with adaptive immune activation through antigen presentation and cytokine signaling, although direct MRONJ-specific evidence for their contribution remains limited. Thus, the immune component of the proposed feedback loop should be interpreted as a multicellular network rather than a macrophage-exclusive pathway. Macrophage polarization was highlighted because MRONJ-specific evidence directly linking macrophage phenotype with lesion progression is comparatively more developed, whereas the roles of neutrophils, lymphocytes and dendritic cells remain less systematically characterized in MRONJ.

### Imbalance of the RANKL/OPG axis and aberrant bone remodeling

#### Inflammatory upregulation of RANKL signaling

The RANKL/RANK/OPG axis is a central osteoimmune pathway that regulates osteoclast differentiation, activation and bone resorption [11,64,65]. RANKL binds to RANK on osteoclast precursors and promotes osteoclastogenesis, whereas OPG functions as a soluble decoy receptor that neutralizes RANKL and limits RANKL–RANK interaction [11,64,65]. Under physiological conditions, this regulatory system helps maintain the balance between bone resorption and bone formation. In MRONJ lesions, however, persistent inflammation may disturb this balance and shift the local microenvironment toward abnormal osteoclastogenic signaling.

Pro-inflammatory cytokines produced by M1-like macrophages and other immune cells, particularly TNF-*α*, IL-1β and IL-6, may increase RANKL expression in osteoblast-lineage cells, stromal cells and activated immune cells [34,35,61–64,66]. TNF-*α* and IL-1β can activate NF-κB and MAPK-related pathways, thereby enhancing RANKL-associated signaling and promoting inflammatory bone resorption [64,66]. IL-6 may further amplify this process through inflammatory and STAT3-related signaling [66]. At the same time, OPG production may be insufficient or reduced under chronic inflammatory conditions, resulting in an increased RANKL/OPG ratio [64–66].

Microbial products may further strengthen this osteoimmune imbalance. Lipopolysaccharide and other biofilm-derived components can activate Toll-like receptor/MyD88/NF-κB pathways and enhance the production of inflammatory cytokines that indirectly support RANKL-driven osteoclastogenic signaling [39,58,59,64,66]. Activated T cells and B cells may also contribute to RANKL production during chronic inflammation, linking adaptive immune activation with local bone destruction [64,65]. Therefore, in MRONJ, microbial persistence and immune activation may converge on the RANKL/OPG axis as a key bridge between inflammation and bone metabolic disturbance.

#### Focal osteoclastogenic signaling in an antiresorptive environment

Binding of RANKL to RANK activates downstream NF-κB, MAPK, AP-1 and NFATc1 signaling, all of which are essential for osteoclast differentiation, fusion, survival and resorptive function [11,65–67]. NFATc1 acts as a master transcriptional regulator of osteoclastogenesis and promotes the expression of osteoclast-associated genes involved in fusion, maturation and matrix degradation [67]. In an inflamed MRONJ microenvironment, cytokines and microbial stimuli may amplify these pathways and generate focal osteoclastogenic signals despite systemic antiresorptive therapy [34,35,52,61–66].

This apparent contradiction is important. MRONJ does not represent generalized skeletal bone resorption. Antiresorptive drugs continue to suppress overall bone turnover and impair physiological remodeling [7–11,33,47]. However, within focal inflammatory lesions, microbial stimulation and cytokine production may generate localized osteoclast-related activity, matrix degradation and bone injury [34,35,52,61–66]. Thus, the pathological process may reflect a local mismatch: systemic remodeling is inhibited, while local inflammatory signaling continues to promote tissue damage, impaired repair and structural fragility.

Inflammation may also enhance tissue breakdown through mechanisms beyond classical osteoclast activation. Reactive oxygen species, nitric oxide and matrix-degrading enzymes produced in chronically inflamed tissues may injure the bone matrix, vascular endothelium and surrounding soft tissues [34,35,61–64,66]. These processes can weaken the local reparative environment and contribute to progressive structural damage even when normal bone remodeling remains pharmacologically suppressed.

#### Bone destruction, repair failure and structural progression

If inflammatory signaling persists and the RANKL/OPG balance remains shifted toward osteoclastogenic signaling, local bone destruction may become increasingly uncoupled from effective repair [34,35,61–66]. Osteoclast-related activity, matrix degradation and inflammatory tissue injury may contribute to trabecular disruption, cortical damage and expansion of necrotic bone [34,35,61–66]. At the same time, antiresorptive therapy reduces normal remodeling capacity, making it more difficult to remove nonviable bone and replace it with newly formed tissue [7–11,33,47].

Chronic inflammation can also impair vascular endothelial function and microcirculation, limiting oxygen and nutrient delivery to the lesion [12,13,33,47]. Reduced perfusion may suppress osteoblast function, delay mucosal healing and weaken the coupling between bone resorption and bone formation [12,13,33,47–51]. As exposed and injured bone surfaces increase, bacterial colonization becomes easier, biofilms become more stable and inflammatory signaling is further amplified [24–32,58,59].

In this setting, bone destruction and repair failure reinforce each other. Local inflammatory signaling promotes structural injury, while impaired remodeling and poor vascular support prevent effective resolution [7–13,33–35,47–51,61–66]. This imbalance may help explain how an initially localized inflammatory lesion progresses toward persistent exposed bone and irreversible osteonecrosis.

### The NLRP3 inflammasome and maintenance of chronic inflammation

#### Synergistic activation of the inflammasome by drugs and microbial stimuli

The NLRP3 inflammasome is an important innate immune sensor that links microbial stimulation, cellular stress and inflammatory cytokine maturation [40,41,68]. Its activation is commonly described as a two-step process. The first step is a priming signal, usually induced by pattern-recognition receptor activation. For example, lipopolysaccharide can activate Toll-like receptor 4 and MyD88-dependent NF-κB signaling, thereby upregulating NLRP3, pro-IL-1β and pro-IL-18 expression [39,40,68]. The second step is an activation signal triggered by cellular stress, including potassium efflux, ATP release, mitochondrial dysfunction, reactive oxygen species generation or lysosomal damage [40,68].

In MRONJ-related environments, antiresorptive drugs and microbial stimulation may cooperate to enhance inflammasome activation. Nitrogen-containing bisphosphonates can interfere with the mevalonate pathway and small GTPase prenylation, leading to altered mitochondrial function, oxidative stress and impaired immune-cell regulation [7–9,34–36,41]. At the same time, biofilm-derived microbial products may provide persistent priming through Toll-like receptor/NF-κB pathways [39,58,59,68]. Experimental evidence in a diabetic mouse model of bisphosphonate-related osteonecrosis showed increased NLRP3, caspase-1 and IL-1β expression in macrophages at extraction wounds, and inhibition of NLRP3-related signaling improved oral wound healing and suppressed osteonecrosis formation [41]. These findings support the possibility that inflammasome activation contributes to persistent inflammation and impaired repair in susceptible MRONJ lesions.

After NLRP3 inflammasome assembly, caspase-1 activation promotes maturation and release of IL-1β and IL-18 [40,68]. Caspase-1 can also cleave gasdermin D, generating an *N*-terminal fragment that forms membrane pores and triggers pyroptotic cell death [69,70]. Therefore, the NLRP3–caspase-1 axis may serve as a molecular bridge linking microbial stimulation, inflammatory cytokine amplification and inflammatory cell death in the MRONJ microenvironment. Evidence from other infection-associated inflammatory diseases further supports the capacity of extracellular and mitochondrial DNA released during tissue injury to amplify inflammation through DNA-sensing and inflammasome-related pathways, including AIM2 and cGAS–STING–NLRP3 signaling, with subsequent caspase-1 activation and pyroptosis. However, whether similar cfDNA-mediated mechanisms operate in MRONJ remains to be determined [71].

#### IL-1β amplification and self-sustaining inflammation

IL-1β is a central amplifier of infection-associated and osteoimmune inflammation [41,72]. After release, IL-1β can bind to IL-1 receptors and activate MyD88-dependent NF-κB and MAPK signaling, creating a positive feedback loop that sustains inflammatory gene transcription [72]. In MRONJ lesions, IL-1β may promote additional cytokine production, recruit neutrophils and monocytes, enhance macrophage activation and increase local inflammatory burden [34,35,41,61–63,72].

IL-1β may also connect inflammation with bone metabolic imbalance. Together with TNF-*α* and IL-6, IL-1β can increase RANKL-associated signaling, reduce the effectiveness of OPG-mediated buffering and promote osteoclastogenic pathways under inflammatory conditions [64–66,72]. At the same time, IL-1β-rich inflammation may suppress osteoblast function and weaken bone-forming capacity, thereby shifting the local microenvironment toward injury rather than repair [34,35,64,66,72].

Physiologically, anti-inflammatory mediators and pro-resolution pathways help terminate inflammation after microbial clearance. In MRONJ, however, persistent biofilm stimulation, reduced perfusion, low bone turnover and drug-induced immune alteration may weaken these braking mechanisms [12,13,33–35,47–51,58,59,61–63]. Sustained NF-κB signaling can further increase inflammasome-related gene expression, while IL-1β can reinforce NF-κB and MAPK activation [39,40,68,72]. Once established, this NLRP3–IL-1β–NF-κB loop may help explain why local inflammation persists even after the initial traumatic or infectious trigger has diminished.

### Programmed cell death in inflammatory bone injury

Before discussing specific forms of programmed cell death, it is important to distinguish established biological mechanisms from MRONJ-specific inference. The molecular pathways of pyroptosis and necroptosis are well characterized in inflammatory and tissue-injury settings, and inflammasome-related activation has been demonstrated in MRONJ-associated experimental models. However, direct evidence that pyroptosis or necroptosis acts as a causal driver in human MRONJ remains limited. Therefore, the following discussion should be interpreted as a mechanistically plausible framework that integrates available evidence rather than as a fully established disease pathway.

Chronic inflammation, oxidative stress and metabolic disturbance may induce regulated cell death in several cell types relevant to MRONJ, including immune cells, osteoblasts, osteocytes and vascular endothelial cells [34,35,40,41,68–70,72–74]. Among these mechanisms, pyroptosis and necroptosis are of particular interest because both can release inflammatory intracellular contents and damage-associated molecular patterns, thereby amplifying local inflammation [68–70,72–74]. Direct evidence for these pathways in human MRONJ remains limited, but they provide a biologically plausible link between persistent inflammation and progressive structural tissue injury.

#### Pyroptosis and inflammatory amplification

Pyroptosis is an inflammatory form of regulated cell death that occurs downstream of inflammasome activation and inflammatory caspase signaling [69,70]. In the canonical pathway, caspase-1 cleaves gasdermin D, releasing its *N*-terminal fragment. This fragment forms membrane pores, leading to cell swelling, membrane rupture and release of IL-1β, IL-18 and damage-associated molecular patterns [69,70]. In MRONJ-related lesions, persistent inflammasome activation driven by microbial products, oxidative stress and drug-altered immune responses may increase susceptibility to pyroptosis-related inflammatory injury [34,35,39–41,58,59,68–70,72].

After pyroptosis, macrophages and other involved cells may release IL-1β, IL-18, ATP and other danger signals. These mediators can activate Toll-like receptor and inflammasome-related pathways in neighboring cells, reinforcing local inflammation and expanding tissue injury [68–70,72]. If pyroptosis occurs in osteoblast-lineage cells or osteocytes, it may further compromise bone formation, matrix maintenance and osteocyte-mediated regulation of remodeling. Although this possibility requires further validation in MRONJ-specific models, pyroptosis offers a plausible mechanism by which inflammatory activation may be converted into structural bone damage.

#### Necroptosis and structural destruction

Necroptosis is a regulated form of necrotic cell death mediated mainly by the RIPK1–RIPK3–MLKL pathway [73,74]. In contrast to apoptosis, necroptosis results in membrane disruption and release of intracellular contents, thereby promoting inflammatory signaling [73,74]. This pathway can be favored under conditions of high inflammatory burden, death-receptor stimulation, innate immune activation and insufficient caspase-8-mediated restraint [73,74].

Persistent TNF-*α*, IL-1β and NF-κB signaling in MRONJ lesions may create a permissive environment for necroptosis, particularly in cells exposed to hypoxia, oxidative stress and microbial products [12,13,33–35,39–41,47,58,59,61–63,72–74]. Activation of RIPK3 and phosphorylation of MLKL can promote MLKL oligomerization and membrane disruption, leading to the release of damage-associated molecular patterns [73,74]. If necroptosis involves osteoblasts, osteocytes or endothelial cells, it may reduce bone-forming capacity, impair intraosseous signaling and worsen microvascular function [12,13,33,47,73,74]. Endothelial injury is especially relevant because it may aggravate local ischemia, reduce nutrient delivery and reinforce an inflammation–ischemia–cell death cycle [12,13,33,73,74].

#### Programmed cell death and progression toward persistent structural osteonecrosis

Programmed inflammatory cell death may contribute to the transition from a potentially reversible inflammatory lesion to persistent structural osteonecrosis [68–70,72–74]. Pyroptosis and necroptosis can release inflammatory mediators and damage-associated molecular patterns that re-engage TLR4-related, inflammasome and cytokine pathways [68–70,72–74]. These signals may further activate macrophages, amplify IL-1β-driven inflammation and promote RANKL/OPG imbalance [61–66,72].

The consequences may extend beyond immune activation. Loss of osteoblasts can reduce bone formation, loss of osteocytes can impair the cellular network that coordinates remodeling, and endothelial injury can worsen hypoxia and local perfusion defects [7–13,33,47–51,73,74]. At the same time, antiresorptive therapy limits the physiological removal and replacement of damaged bone [7–11,33,47]. When inflammation, cell death and repair failure reinforce one another, tissue-repair capacity may progressively decline and necrotic bone may become less likely to be replaced by viable bone.

In this context, programmed cell death should be viewed as a potential downstream amplifier rather than a proven primary cause of MRONJ. Its main relevance lies in explaining how persistent microbial and inflammatory stimulation may be translated into persistent structural damage. Future MRONJ studies should therefore assess pyroptosis- and necroptosis-related markers in longitudinal human samples and disease-specific experimental models to determine whether these pathways represent therapeutic targets or markers of advanced tissue injury. Together, macrophage polarization, inflammatory cytokine production, RANKL/OPG imbalance, inflammasome activation and programmed cell death may form an immune–bone metabolic amplification axis that links persistent inflammation with structural tissue injury in MRONJ (Figure 3).

**Figure 3.**
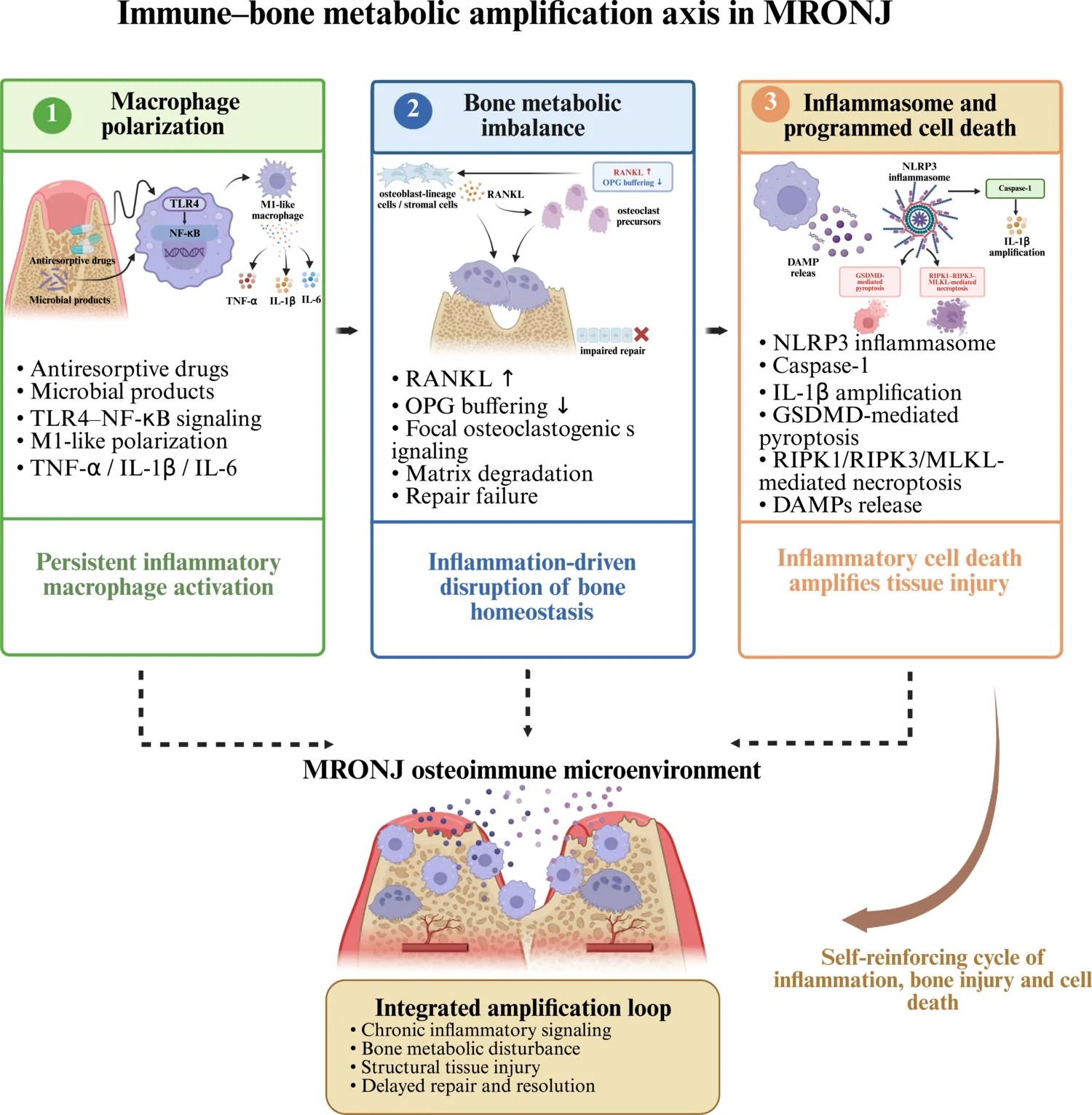
Immune–bone metabolic amplification axis in MRONJ. This schematic summarizes the immune and bone metabolic amplification mechanisms proposed to sustain MRONJ lesions. Antiresorptive drugs and microbial products may promote TLR4–NF-κB signaling, M1-like macrophage polarization and persistent release of inflammatory cytokines, including TNF-*α*, IL-1β and IL-6. These inflammatory mediators may disturb the RANKL/OPG axis, enhance focal osteoclastogenic signaling, promote matrix degradation and impair tissue repair. In parallel, NLRP3 inflammasome activation, caspase-1-dependent IL-1β amplification, GSDMD-mediated pyroptosis and RIPK1–RIPK3–MLKL-mediated necroptosis may contribute to inflammatory cell death and DAMP release. Together, these processes form an integrated amplification loop linking chronic inflammatory signaling, bone metabolic disturbance, structural tissue injury and delayed repair in the MRONJ osteoimmune microenvironment.

### An integrated mechanistic model and phase-progression hypothesis

Taken together, microbial dysbiosis, immune dysregulation, disturbed bone remodeling and regulated cell death suggest that medication-related osteonecrosis of the jaw is a multistep disorder driven by interacting pathological processes rather than by a single mechanism [21–32,34–41,52–56,58,59,61–70,72–74]. We therefore propose an integrated osteoimmuno-microbial feedback model to explain how a drug-induced susceptible jawbone niche may progress from local vulnerability to persistent structural osteonecrosis. In this framework, microbial persistence, biofilm maturation, inflammatory amplification, RANKL/OPG imbalance and programmed cell death form interconnected positive feedback loops that progressively increase the local pathological burden. The proposed process involves overlapping phases, including local vulnerability, microbial colonization, biofilm maturation, inflammatory amplification, bone metabolic imbalance, programmed cell death and structural repair failure. Systemic host factors, including diabetes, smoking and concomitant immunosuppressive therapy, may further modify this trajectory by impairing vascular support, mucosal repair and antimicrobial immune clearance, thereby lowering the threshold for feedback-loop persistence and progression.

Antiresorptive and antiangiogenic therapies may first create a low-turnover, poorly vascularized and poorly regenerative microenvironment by suppressing osteoclast-mediated remodeling, impairing angiogenesis, delaying mucosal repair and altering local immune defense [7–13,33–38,47–51]. In this permissive niche, oral microbial communities may shift toward dysbiosis, with enrichment of anaerobic and periodontal-associated organisms and development of polymicrobial biofilms [21–32,46,52–56,58,59]. Persistent biofilms can provide continuous microbial stimulation and activate TLR4–NF-κB signaling, NLRP3 inflammasome pathways and IL-1β-dependent inflammatory amplification [39–41,58,59,68,72]. These inflammatory signals may promote M1-like macrophage polarization, disturb the RANKL/OPG axis and generate focal osteoclastogenic and matrix-degrading activity despite systemic suppression of bone remodeling [52,61–67]. For conceptual clarity, macrophage polarization is depicted as a representative immune axis in the model; however, this simplification does not exclude contributions from neutrophils, T cells, dendritic cells and other immune populations. In later phases, pyroptosis, necroptosis and damage-associated molecular pattern release may further amplify inflammation, impair osteoblast, osteocyte and endothelial function, and reduce tissue-repair capacity [68–70,72–74].

#### Phase-wise interpretation of the proposed model

The proposed phase-wise model should be interpreted as a conceptual framework rather than as a rigid clinical staging system. The phases are likely to overlap, and different pathological axes may dominate at different time points or in different patients.

#### Phase I: susceptible jawbone microenvironment

Prolonged exposure to bisphosphonates, denosumab, antiangiogenic agents or other targeted therapies may suppress bone remodeling, impair local vascular support and delay mucosal repair [7–13,33,47–51]. Reduced osteoclast-mediated turnover limits physiological repair of microdamage, while impaired angiogenesis and reduced perfusion weaken oxygen and nutrient delivery. At the same time, epithelial and fibroblast dysfunction may compromise mucosal barrier integrity. Together, these changes create a low-turnover, poorly regenerative and immunologically vulnerable niche.

#### Phase II: microbial dysbiosis and biofilm establishment

After mucosal disruption caused by tooth extraction, periodontal inflammation, peri-apical disease or other local trauma, the altered jawbone niche may permit microbial colonization and ecological remodeling [17–20,47–51]. Anaerobic and periodontal-associated organisms, including Actinomyces-associated bacteria, Fusobacterium nucleatum, Porphyromonas gingivalis and related pathobionts, may expand within exposed or injured tissues [21–32,52–56]. Rather than reflecting a single-pathogen infection, this phase is better understood as polymicrobial restructuring and biofilm maturation in a susceptible host environment [52–56,58,59].

#### Phase III: inflammatory amplification and immune dysregulation

Once biofilms mature, microbial products such as lipopolysaccharide, lipoproteins, extracellular DNA and outer-membrane vesicles can provide persistent innate immune stimulation [39,58,59]. These signals may activate TLR4–NF-κB pathways, promote NLRP3 inflammasome activation and increase IL-1β release [39,41,68,72]. In parallel, macrophages may remain biased toward an M1-like inflammatory phenotype, while M2-like reparative responses and inflammatory resolution are insufficient [34,35,61–63]. This phase connects microbial persistence with sustained osteoimmune activation.

#### Phase IV: bone metabolic imbalance and structural injury

Chronic inflammatory signaling may disturb the RANKL/OPG axis and generate focal osteoclastogenic and matrix-degrading activity [64–67]. This does not mean that MRONJ is driven by generalized bone resorption. Instead, it suggests a local mismatch: systemic bone turnover is pharmacologically suppressed, while focal inflammatory lesions continue to experience cytokine-driven tissue injury, impaired repair and structural weakening [7–11,33,47,52,61–67]. Programmed cell death pathways, including pyroptosis and necroptosis, may further damage osteoblasts, osteocytes, endothelial cells and immune cells, thereby reducing local regenerative capacity [68–70,72–74].

#### Phase V: persistent structural osteonecrosis

When microbial persistence, inflammatory amplification, bone metabolic imbalance and programmed cell death become self-reinforcing, local repair capacity may progressively decline. Reduced perfusion, impaired bone remodeling, matrix degradation and persistent biofilm stimulation may prevent replacement of necrotic bone and closure of exposed surfaces [7–13,21–32,47–56,58,59,64–70,72–74]. At this phase, the lesion may cross a functional threshold beyond which spontaneous resolution becomes unlikely and structural osteonecrosis becomes persistent.

Overall, this model proposes that MRONJ develops through interacting positive feedback loops rather than through a single linear pathway. Biofilms sustain microbial stimulation; inflammasome activation and IL-1β signaling amplify inflammation; RANKL/OPG imbalance links immune activation to bone metabolic disturbance; and programmed cell death converts inflammatory stress into structural tissue injury. DAMPs released from damaged cells may then reactivate TLR4-related and inflammasome pathways, maintaining the loop [68–70,72–74]. The central hypothesis is that medication exposure lowers the buffering and reparative capacity of jawbone tissue, allowing infection- and inflammation-related stressors that are normally controllable to become self-sustaining and progressively destructive. This conceptual phase-wise model suggests that overlapping pathological processes progressively increase the local pathological burden. Once this burden exceeds local reparative capacity, MRONJ lesions may cross a functional threshold and progress from a potentially reversible local imbalance to persistent structural osteonecrosis (Figure 4).

#### Testable predictions derived from the model

The proposed feedback model generates at least two testable predictions. First, if microbial dysbiosis participates in disease propagation before overt structural breakdown, longitudinal sampling of at-risk patients should detect reproducible shifts in oral microbial community composition and associated inflammatory signals before the onset of clinically exposed bone. Patients with persistent preclinical dysbiosis would be expected to have a higher probability of subsequent MRONJ development than those without such changes. Second, if impaired host microbial clearance is an important component of the feedback loop, reduced innate immune function should be associated with greater biofilm persistence and more pronounced inflammatory and structural progression. This prediction could be tested by relating neutrophil or macrophage functional measures to microbial burden and clinical progression in longitudinal cohorts, and by experimentally manipulating host clearance capacity in preclinical models. These predictions are hypotheses generated by the present framework and require prospective validation.

**Figure 4.**
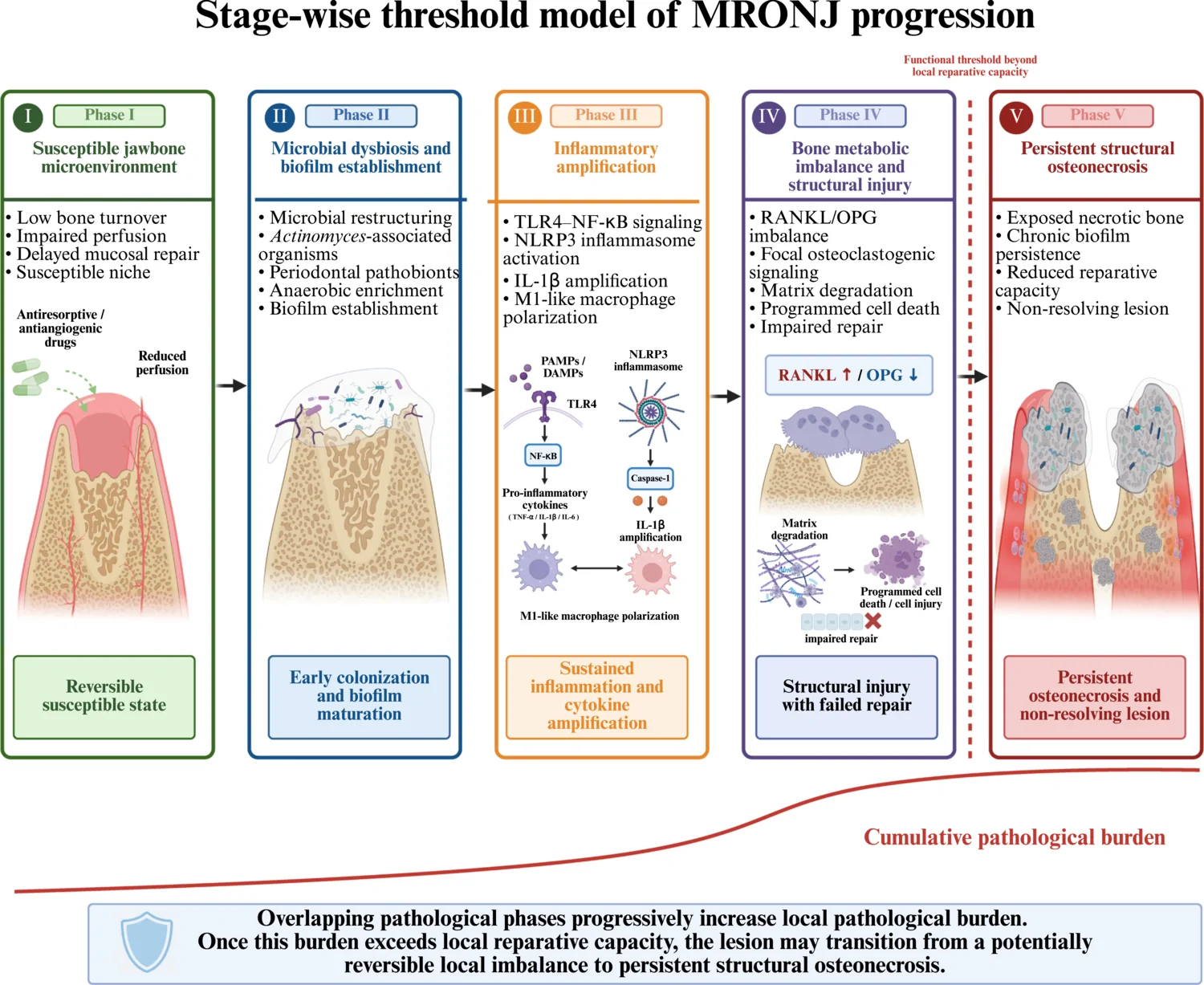
Phase-wise threshold model of MRONJ progression. This schematic illustrates a conceptual phase-wise model in which MRONJ progresses from a drug-induced susceptible jawbone microenvironment to persistent structural osteonecrosis. In the early phases, suppressed bone turnover, impaired perfusion and delayed mucosal repair create a permissive niche for microbial dysbiosis and biofilm establishment. Persistent biofilm stimulation may then activate TLR4–NF-κB signaling, NLRP3 inflammasome pathways, IL-1β amplification and M1-like macrophage polarization. As inflammatory signaling persists, RANKL/OPG imbalance, focal osteoclastogenic signaling, matrix degradation, programmed cell death and impaired repair may increase local structural injury. When the cumulative pathological burden exceeds local reparative capacity, the lesion may cross a functional threshold and progress toward persistent structural osteonecrosis. MRONJ, medication-related osteonecrosis of the jaw; TLR4, Toll-like receptor 4; NF-κB, nuclear factor-κB; NLRP3, nucleotide-binding oligomerization domain-like receptor protein 3; IL-1β, interleukin-1β; RANKL, receptor activator of nuclear factor-κB ligand; OPG, osteoprotegerin; DAMPs, damage-associated molecular patterns.

## Clinical implications and intervention strategies: from pathological loops to targeted disruption

The integrated model proposed above suggests that management of medication-related osteonecrosis of the jaw may need to extend beyond infection control and local debridement alone. If lesion progression is sustained by interconnected osteoimmuno-microbial feedback loops, therapeutic strategies should aim to interrupt the dominant amplification processes active at a given disease phase. From this perspective, a phase-adapted and multidimensional approach may be more appropriate than reliance on a single intervention. The following sections discuss potential intervention axes involving microbial control, inflammatory modulation, restoration of bone remodeling balance, preservation of tissue vitality and future precision management.

### Microecological intervention: limiting microbial persistence and biofilm establishment

Microbial restructuring and biofilm maturation may represent early and persistent contributors to MRONJ progression [21–32,52–56,58,59]. Once a stable polymicrobial biofilm is established, inflammatory activation, impaired bone remodeling and cell-death-associated tissue injury may become more difficult to reverse [58,59,68–70,72–74]. Therefore, strategies that reduce microbial burden, prevent biofilm maturation and support ecological balance are central to prevention and early management.

#### Preventive oral management and reduction of local triggers

Current clinical guidance emphasizes oral assessment, elimination of active dental infection and optimization of oral hygiene before or during antiresorptive or antiangiogenic therapy, especially in high-risk patients [2,3,75]. Treatment of periodontitis, control of peri-apical infection, reduction of traumatic prosthetic irritation and avoidance of unnecessary invasive procedures may reduce local inflammatory load and limit bacterial entry into a low-turnover, poorly repaired jawbone niche [2,3,17–20,75]. When dentoalveolar surgery is unavoidable, atraumatic technique, infection control, primary soft tissue closure when appropriate and close postoperative surveillance may reduce the probability of persistent bone exposure [2,3,75]. These measures are most relevant before disease onset or during early local vulnerability, when the pathological feedback loops may still be preventable.

#### Anti-biofilm strategies and local microbial control

Mature biofilms are inherently difficult to eradicate because their extracellular polymeric matrix limits antimicrobial penetration, protects deeper microorganisms from host immune attack and supports phenotypic heterogeneity [58,59]. Therefore, increasing systemic antibiotic exposure alone may not fully disrupt MRONJ-associated biofilms and may increase adverse effects or antimicrobial resistance [32,58,59]. More targeted approaches may include local antimicrobial delivery, biofilm-disrupting agents, matrix-degrading enzymes, antimicrobial peptides and nanocarrier-based systems designed to improve drug penetration into biofilm communities [58,59,76].

Stimuli-responsive nanocarriers are of particular conceptual interest because they can respond to biofilm-associated conditions such as pH changes, bacterial enzymes and hypoxia, thereby enhancing antimicrobial accumulation and release within biofilms [76]. Recent evidence from other microbiota-associated diseases also illustrates the potential of simultaneously targeting pathogenic microorganisms and the host immune microenvironment. For example, targeted elimination of Fusobacterium nucleatum using a nanotherapeutic platform in colorectal cancer not only reduced the pathogenic bacterial burden but also remodeled local immune responses. Although this strategy has not been evaluated in MRONJ, it provides a cross-disease proof of concept for future approaches that integrate local microbial control with immune modulation [77].

However, these approaches remain largely preclinical or early translational in oral biofilm research, and their specific efficacy and safety in MRONJ have not been established. In MRONJ, anti-biofilm therapy should therefore be viewed as a potential adjunct to mechanical debridement, local wound care and systemic management rather than as a replacement for established clinical treatment.

#### Microecological reconstruction and colonization resistance

Microecological intervention should not be interpreted as simple sterilization. The oral microbiota contains commensal communities that contribute to epithelial barrier integrity, immune balance and colonization resistance [42–46]. Experimental evidence suggests that indigenous microbiota may have protective effects against MRONJ development under specific conditions [46]. Therefore, future strategies should not only suppress pathogenic biofilms but also preserve or restore a stable oral ecosystem. Probiotics, postbiotics and other microbiota-modulating approaches may eventually support ecological resilience, but their role in MRONJ remains experimental and requires disease-specific validation.

### Inflammatory modulation: restoring resolution rather than broad immunosuppression

Persistent inflammatory amplification may represent a key transition point between microbial colonization and progressive structural injury in medication-related osteonecrosis of the jaw. Biofilm-derived products and drug-altered immune responses may activate Toll-like receptor–nuclear factor-κB signaling, nucleotide-binding oligomerization domain-like receptor protein 3 inflammasome pathways and interleukin-1β release, thereby sustaining macrophage activation and inflammatory cytokine production. From a therapeutic perspective, the aim should not be broad suppression of host immunity, because effective microbial clearance remains essential in biofilm-rich lesions. Instead, future strategies should focus on restoring inflammatory resolution, limiting excessive cytokine amplification and preserving antimicrobial defense.

Potential intervention points include modulation of Toll-like receptor–nuclear factor-κB signaling, attenuation of nucleotide-binding oligomerization domain-like receptor protein 3–interleukin-1β amplification, regulation of macrophage polarization and promotion of pro-resolution immune responses. These approaches remain investigational in medication-related osteonecrosis of the jaw and should be interpreted as mechanism-informed research directions rather than established clinical treatments. Their value may be greatest in intermediate phases, when microbial persistence and inflammatory amplification are established but extensive structural tissue loss has not yet become irreversible.

### Restoring bone remodeling balance: beyond further suppression of resorption

The RANKL/OPG axis links inflammatory signaling to bone remodeling disturbance in the proposed model. Pro-inflammatory cytokines and microbial stimulation may increase RANKL-associated signaling and reduce OPG-mediated buffering, thereby generating focal osteoclastogenic and matrix-degrading activity [39,58,59,64–66]. However, MRONJ occurs in a pharmacologically antiresorptive environment. The pathological issue is therefore not generalized bone resorption, but a local mismatch in which inflammatory tissue injury progresses while physiological remodeling and repair remain suppressed [7–11,33–35,47,61–66].

#### Modulating osteoclastogenic signaling without further over-suppression

Because excessive or prolonged suppression of RANKL signaling can further reduce bone turnover, therapeutic strategies should not simply aim to intensify antiresorptive effects [7–11,33,65]. A more rational approach may be to reduce upstream inflammatory signals, including IL-1β, TNF-*α* and persistent microbial stimulation, thereby indirectly lowering focal RANKL-driven injury while preserving the capacity for physiological repair [39,58,59,61–66,72]. Local or time-limited modulation may theoretically be preferable to chronic systemic suppression, but this concept requires experimental and clinical validation.

#### Supporting osteogenesis, angiogenesis and tissue repair

Progression toward irreversible osteonecrosis is associated with loss of reparative capacity. Chronic inflammation, ischemia, biofilm persistence and programmed cell death can impair osteoblast function, endothelial integrity and matrix renewal [12,13,33–35,47–51,58,59,69,70,72–74]. Strategies that support angiogenesis, improve microcirculation, enhance oxygen and nutrient delivery, and preserve osteoblast and osteocyte viability may therefore complement infection control and inflammatory modulation. Clinically, this reinforces the importance of soft tissue closure, local wound stability, reduction of traumatic irritation and close follow-up [2,3,75]. Mechanistically, it highlights the need to restore the coupling between vascular support, bone formation and immune resolution.

### Limiting programmed cell death: a potential strategy for structural preservation

In the proposed model, inflammatory programmed cell death may convert persistent immune activation into structural tissue injury [68–70,72–74]. Pyroptosis and necroptosis can release damage-associated molecular patterns, amplify Toll-like receptor and inflammasome signaling, and further impair osteoblast, osteocyte and endothelial function [68–70,72–74]. Therefore, limiting inflammatory cell-death pathways could theoretically reduce tissue breakdown and preserve residual reparative capacity. At present, however, this remains a mechanism-based and investigational strategy in MRONJ.

#### The inflammasome–GSDMD axis and pyroptotic injury

Pyroptosis requires inflammatory caspase-mediated cleavage of gasdermin D, resulting in membrane pore formation, IL-1β release and damage-associated molecular pattern release [69,70]. Because pyroptosis is linked to the NLRP3–caspase-1–IL-1β pathway, interventions that reduce excessive inflammasome activation may also limit pyroptotic spread [40,41,68–70,72,78]. However, pyroptosis contributes to host defense against infection, and broad inhibition could be harmful in biofilm-rich lesions [58,59,68,78]. Future studies should determine whether selective, stage-specific modulation of this axis can reduce tissue injury without impairing microbial clearance.

#### The RIPK1/RIPK3/MLKL pathway and necroptosis-associated damage

Necroptosis is mainly mediated by the RIPK1/RIPK3/MLKL pathway and results in membrane rupture and inflammatory intracellular content release [73,74,79]. In MRONJ, persistent TNF-*α*, IL-1β, hypoxia and microbial products may create a permissive environment for necroptosis, particularly in cells exposed to severe metabolic and inflammatory stress [12,13,33–35,39–41,47,58,59,61–63,72–74]. If necroptosis affects osteoblasts, osteocytes or endothelial cells, it may reduce bone formation, impair intraosseous cellular signaling and worsen microvascular compromise [12,13,33,47,73,74]. Modulating this pathway may therefore represent a future strategy for limiting structural damage, but direct MRONJ-specific evidence is still lacking [79].

#### From anti-inflammation alone to structural preservation

In advanced MRONJ, tissue deterioration may continue even when inflammation is partially controlled because the lesion has already lost sufficient reparative capacity [7–13,33,47–51,68–70,72–74]. Treatment priorities may therefore change over time. Early-stage intervention should emphasize prevention of microbial colonization, biofilm control and inflammatory resolution [2,3,21–32,58,59,75]. Later-stage intervention may also need to address structural preservation, soft tissue closure, vascular support and maintenance of viable bone [2,3,12,13,33,47–51,75]. This shift from anti-inflammation alone toward protection of tissue vitality is consistent with a stage-tailored model of MRONJ progression.

### Phase-adapted intervention framework

MRONJ appears to evolve through overlapping pathological phases, ranging from local tissue vulnerability and microbial colonization to inflammatory escalation, repair failure and structural osteonecrosis. Management should therefore not be considered a fixed strategy that applies uniformly across all phases. Instead, intervention may need to be adapted to the dominant pathological processes present at a given phase. This concept should be interpreted as a mechanism-informed framework rather than as a validated clinical staging or treatment algorithm. To make the phase-adapted strategy more explicit, each proposed pathological phase can be linked to a dominant therapeutic target and intervention priority. In the earliest phases, the main goal is to preserve mucosal integrity, reduce local trauma and prevent microbial establishment. Once dysbiosis and biofilm persistence become established, microbial burden and biofilm control become increasingly important. During inflammatory amplification, intervention may need to address both persistent microbial stimulation and excessive host inflammatory signaling. When bone metabolic imbalance and repair failure become prominent, preservation of vascular support, osteogenic capacity and tissue viability becomes a greater priority. In advanced structural osteonecrosis, removal of necrotic bone, control of infection and reconstruction of a stable soft-tissue environment become central. These phases may overlap, and the proposed interventions should be interpreted according to the dominant pathological process rather than as a rigid treatment sequence. The proposed phase-adapted intervention framework is summarized in Table 1.

**Table 1.** Phase-adapted intervention framework for MRONJ.

Proposed phase	Dominant pathological process	Main intervention target	Phase-adapted strategy
Phase I: susceptible jawbone niche	Low turnover, impaired mucosal/vascular repair	Local trauma and barrier integrity	Oral assessment, treatment of active dental infection, oral hygiene optimization, avoidance of unnecessary trauma, atraumatic procedures
Phase II: dysbiosis and biofilm establishment	Microbial restructuring and biofilm persistence	Microbial burden and biofilm	Infection control, local wound care, mechanical biofilm disruption; targeted anti-biofilm approaches remain investigational
Phase III: inflammatory amplification	Persistent inflammatory signaling	Microbial stimulation + excessive inflammation	Continue microbial control; investigate TLR4–NF-κB, NLRP3–IL-1β and macrophage-polarization modulation
Phase IV: bone/repair imbalance	Bone metabolic disturbance, vascular and structural injury	Repair capacity and tissue vitality	Reduce inflammatory burden; support osteogenesis, angiogenesis, soft-tissue repair and structural preservation
Phase V: persistent structural osteonecrosis	Necrotic bone as a persistent inflammatory/biofilm reservoir	Necrotic tissue and non-healing wound	Debridement/resection in selected patients, infection control, soft-tissue closure, wound care and multidisciplinary support

#### Phases I–II: microecological control and trauma management

In the earliest phases, lesions may remain preventable, reversible or locally limited. Management should focus on minimizing avoidable traumatic procedures, optimizing periodontal and peri-apical health, improving oral hygiene and reducing local factors that promote microbial colonization and biofilm formation [2,3,17–20,75]. For patients receiving or scheduled to receive antiresorptive, antiangiogenic or other MRONJ-associated therapies, systematic oral assessment and elimination of active dental infection remain central preventive measures [2,3,75]. When dentoalveolar procedures are unavoidable, atraumatic technique, infection control, preservation of mucosal healing and close postoperative surveillance are particularly important [2,3,75]. These measures aim to prevent microbial persistence and biofilm maturation before self-sustaining inflammatory loops become established.

#### Phase III: attenuation of inflammatory amplification

Once biofilms and inflammatory networks become self-sustaining, antimicrobial therapy alone may be insufficient to halt disease progression [32,58,59]. At this phase, intervention may need to address not only microbial burden but also inflammatory amplification. Potential mechanistic targets include TLR4–NF-κB signaling, the NLRP3–IL-1β axis and macrophage polarization [39–41,58,59,61–63,68,72,78]. However, these approaches remain investigational in MRONJ. The therapeutic goal should be framed as restoration of inflammatory resolution rather than broad immunosuppression, because excessive suppression of innate immunity may impair microbial clearance in biofilm-rich lesions [58,59,68,78].

#### Phase IV: combined regulation of bone remodeling and structural injury

When bone metabolic imbalance, matrix degradation and programmed cell death become prominent, multi-axis intervention may become increasingly important [12,13,33–35,47–51,58,59,61–70,72–74]. Reducing inflammatory burden may indirectly attenuate focal RANKL-associated signaling, whereas strategies that support osteogenesis, angiogenesis and soft tissue repair may help preserve residual regenerative capacity [12,13,33–35,47–51,64–70,72–74]. Pyroptosis- and necroptosis-related pathways may also contribute to structural tissue injury, but direct MRONJ-specific therapeutic evidence remains limited [68–70,72–74]. Therefore, modulation of programmed cell death should currently be regarded as a research direction rather than an established treatment strategy.

#### Phase V: debridement and multi-axis supportive management

In advanced disease, necrotic bone may become a persistent structural reservoir for biofilm formation and chronic inflammation [21–32,58,59]. Surgical debridement or resection may be required in selected patients, particularly when necrotic bone prevents mucosal closure or sustains infection [2,3,75]. However, surgery alone may not fully address the biological loops that drive recurrence, delayed healing or persistent inflammation [2,3,21–32,58,59,75]. Local wound care, infection control, soft tissue closure, support of the reparative microenvironment and multidisciplinary management may therefore be required [2,3,75]. In this phase, the goal is not only to remove necrotic tissue but also to reduce ongoing microbial, inflammatory, vascular and metabolic stress.

#### Rationale for multi-axis intervention

MRONJ is unlikely to be sustained by a single abnormal pathway. Once microbial persistence, inflammatory amplification, bone remodeling imbalance and cell-death-associated tissue injury become interconnected, targeting only one pathway may have limited benefit. A sequential and multidimensional strategy may therefore be more suitable, especially when it addresses the dominant amplification mechanisms active at different disease phases. This concept provides a rationale for integrating preventive dentistry, infection control, immune modulation, tissue-repair support and surgical management within a phase-adapted framework.

### Future perspectives: multi-omics integration and risk stratification

Advances in microbiomics, immunomics, transcriptomics, proteomics, metabolomics and computational modeling may help shift MRONJ research from single-factor explanations toward network-based disease characterization [80–87]. At present, however, multi-omics evidence in MRONJ remains fragmented, and many studies are exploratory, cross-sectional or limited by small sample size [80–83]. Future research should therefore emphasize longitudinal sampling, multicenter validation and integration with clinical and imaging data.

#### Multi-omics characterization of pathological networks

Microbiome analysis can identify dysbiotic microbial communities and potential loss of colonization resistance, whereas salivary and tissue proteomics may reveal inflammatory, immune and wound-healing-related biomarkers [21–32,52,80,81]. Bioinformatic analyses have begun to identify candidate pathways and molecular signatures associated with MRONJ susceptibility or progression [82]. Genetic and pharmacogenomic studies may further clarify why only a subset of exposed patients develop MRONJ, although current evidence remains insufficient for routine clinical prediction [83]. Integrating microbial, immune, genetic, proteomic and metabolic data may provide a more comprehensive view of the pathological network underlying MRONJ. More specifically, multi-omics approaches in MRONJ should address several unresolved disease-specific questions. First, longitudinal integration of microbiome, transcriptomic, proteomic and metabolomic data may help determine whether microbial dysbiosis precedes clinical bone exposure or mainly emerges after tissue necrosis. Second, multi-omics profiling may clarify why only a subset of patients exposed to the same MRONJ-associated medications develop disease, by identifying host–microbial signatures associated with susceptibility. Third, integrated molecular profiling may help define the biological transition from a potentially reversible inflammatory lesion to persistent structural osteonecrosis and identify biomarkers associated with progression, healing failure or recurrence.

#### Risk stratification and early prediction

Future risk models should move beyond medication exposure alone. A more informative strategy may combine drug-related variables, dental risk factors, systemic comorbidities, local inflammatory burden, oral microbial status, host immune phenotype, genetic susceptibility and imaging features [2,3,16–20,75,83–87]. Such models may help identify patients with pronounced microbial dysbiosis, exaggerated inflammatory responses or weakened reparative capacity before persistent structural tissue injury occurs. However, risk stratification tools must be validated in large multicenter cohorts before they can guide clinical decision-making.

#### Artificial intelligence and dynamic monitoring

Artificial intelligence may support MRONJ research by identifying complex patterns across clinical variables, imaging data and high-dimensional biological datasets [84–87]. Recent studies suggest that machine-learning models, pharmacovigilance databases and radiomics features may have potential for MRONJ risk assessment, drug-risk prediction or classification [84–87]. Radiomics analysis of panoramic radiographs, for example, has been explored as a method for identifying imaging patterns associated with MRONJ risk or classification [87]. Nevertheless, current artificial intelligence studies are limited by small datasets, retrospective designs, heterogeneous outcome definitions and insufficient external validation [84–87]. Future models should prioritize transparency, interpretability, standardized endpoints and clinical usability. For MRONJ, the major value of artificial intelligence may lie in integrating heterogeneous clinical, imaging and biological information to address specific prediction tasks. These include identifying high-risk patients before clinical bone exposure, predicting lesion progression or failure of conservative management, and estimating healing or recurrence risk after treatment. Future models may combine medication-related variables, dental and systemic risk factors, imaging or radiomic features, and, where available, microbiome or immune biomarkers. Importantly, such models should be evaluated using clinically meaningful and standardized endpoints and validated in independent, preferably multicenter and longitudinal cohorts.

#### From single-pathway treatment to network regulation

Future MRONJ management is unlikely to depend on inhibition of one isolated pathway. A more realistic goal is to identify the dominant pathological nodes in each patient and stabilize the disease network through phase-specific, multi-axis intervention. Improved risk stratification, multi-omics profiling, artificial intelligence-assisted prediction and longitudinal monitoring may eventually support earlier intervention before lesions cross the threshold from reversible local imbalance to persistent structural osteonecrosis. For now, these approaches should be presented as promising research directions rather than established clinical standards. Accordingly, future MRONJ management may benefit from a phase-adapted, multi-axis framework that integrates microbial control, inflammatory modulation, repair support, tissue preservation, multi-omics profiling, imaging/radiomics, artificial intelligence-assisted risk prediction and longitudinal monitoring (Figure 5).

**Figure 5.**
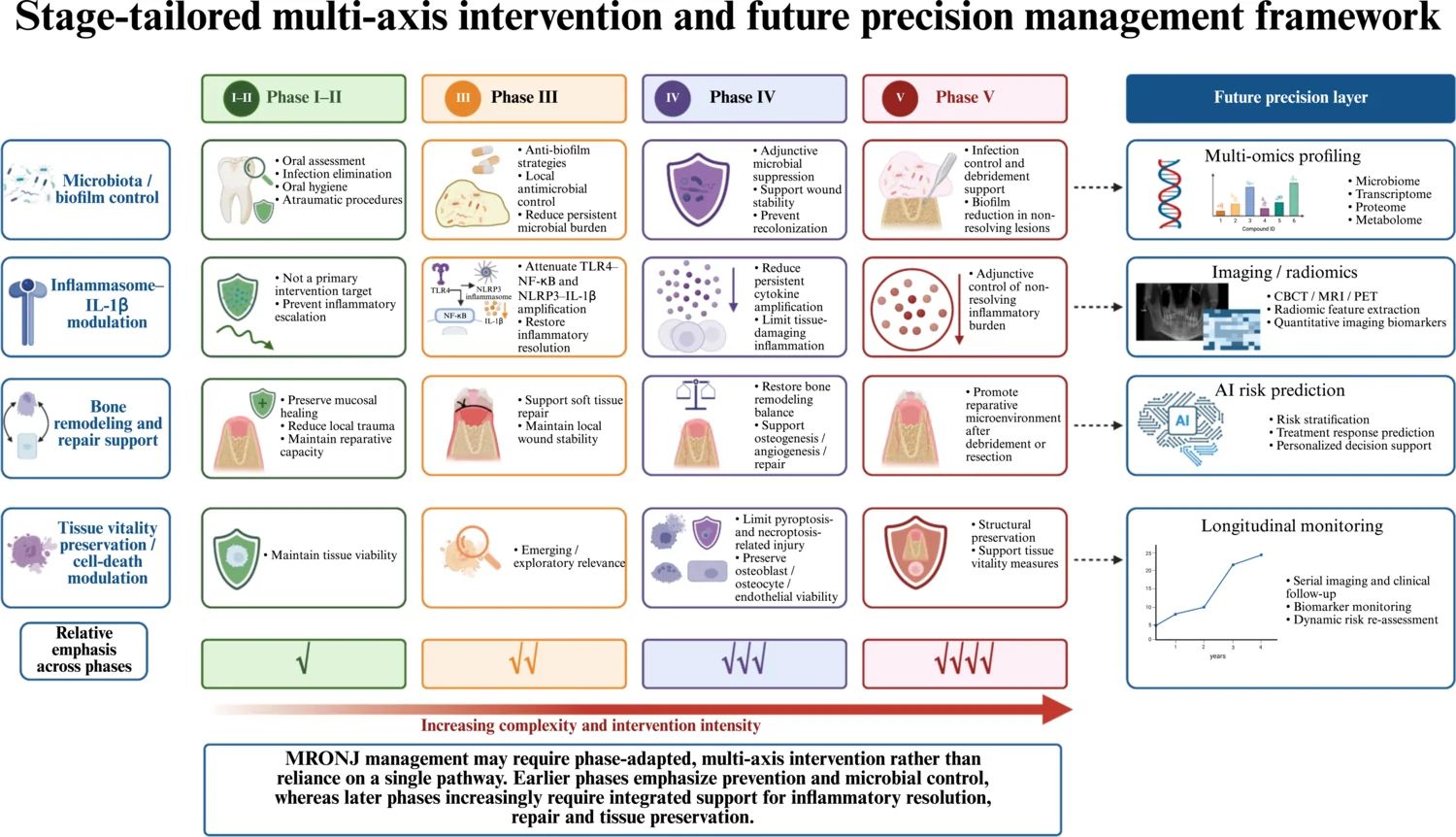
Phase-adapted multi-axis intervention and future precision management framework. This schematic summarizes a phase-adapted intervention framework derived from the proposed osteoimmuno-microbial feedback model of MRONJ. Early phases emphasize oral assessment, infection elimination, oral hygiene optimization, atraumatic procedures and prevention of microbial colonization. As biofilm maturation and inflammatory amplification become established, intervention may need to address both microbial burden and inflammatory signaling, including TLR4–NF-κB and NLRP3–IL-1β pathways. In later phases, restoration of bone remodeling balance, support of osteogenesis and angiogenesis, soft tissue repair, preservation of tissue vitality and structural protection become increasingly important. Multi-omics profiling, imaging/radiomics, artificial intelligence-assisted risk prediction and longitudinal monitoring may support future precision management. This framework should be interpreted as a mechanism-informed perspective rather than a validated clinical treatment algorithm.

## Research limitations and key controversies

Although understanding of medication-related osteonecrosis of the jaw (MRONJ) has advanced substantially, several important controversies and unresolved questions remain. The osteoimmuno-microbial feedback model proposed in this Review should therefore be interpreted as a conceptual framework rather than as a validated causal pathway or clinical staging system.

Additional clinical, experimental and mechanistic literature further illustrates the breadth of evidence that informs this framework. Earlier clinical reviews, task force reports and cohort or case-control studies have characterized major risk factors and clinical patterns of bisphosphonate- or medication-related osteonecrosis of the jaw [88–93]. Systematic reviews and evidence syntheses have also highlighted the heterogeneity and limitations of available treatment strategies [94,95]. Experimental animal models have provided important insights into how antiresorptive exposure, local inflammation and periapical disease may interact to produce osteonecrosis-like jaw lesions [96–99]. In parallel, foundational studies of oral microbial complexes, dental plaque biofilms, biofilm matrix biology and anti-biofilm therapeutic strategies help contextualize microbial persistence in MRONJ [100–103]. Core concepts from osteoimmunology, RANKL/OPG biology, inflammasome signaling and regulated cell death further support the biological plausibility of the proposed immune–bone–cell death axes [104–111]. More broadly, concepts from radiomics and high-performance medicine support the use of quantitative imaging features and artificial intelligence-assisted analysis to improve individualized prediction, dynamic monitoring and clinical decision support [112–114].

First, many findings related to microbial dysbiosis, inflammasome activation, macrophage polarization and programmed cell death are derived from animal models, in vitro experiments or small clinical studies [28–32,34,35,41,52,61–63,68–70,72–74]. These studies are valuable for identifying candidate mechanisms and biological associations, but they cannot fully reproduce the complexity of human MRONJ. Differences between experimental models and clinical disease may involve drug type, dosage, route and duration of administration, systemic comorbidities, oral microbial exposure, local trauma, jawbone biology and host immune status. Therefore, mechanistic findings should not be directly translated into clinical management without validation in well-designed human studies.

Second, most clinical evidence remains cross-sectional or retrospective, which limits causal interpretation [28–32,52,80–87]. This limitation is particularly relevant to microbiome studies, many of which analyze samples collected after bone exposure or necrosis has already occurred [28–32,52]. Under these conditions, it is difficult to determine whether observed microbial changes are early drivers, adaptive responses to a drug-altered niche or secondary colonizers of necrotic bone. Prospective longitudinal studies with sampling before treatment, after local trauma and during lesion development are needed to clarify whether microbial dysbiosis contributes to early pathogenesis, lesion persistence or both.

Third, the role of microorganisms in MRONJ remains debated. One view is that infection is mainly a secondary amplifier, whereas suppressed bone remodeling, impaired angiogenesis and delayed mucosal repair represent the primary pathological substrate [2–13,21–24,33,47–51]. Another view is that local infection, periodontal inflammation and biofilm formation may participate earlier in disease development, especially after mucosal injury or dentoalveolar procedures [17–32,46,52]. Experimental evidence also suggests that commensal microbial communities may have protective effects under some conditions [46]. Together, these observations indicate that microbial involvement in MRONJ is unlikely to be uniform. Its role may vary according to disease phase, medication exposure, host immunity, microbial community structure and local tissue conditions.

Fourth, several intervention strategies discussed in this Review remain hypothetical or early translational. Approaches targeting biofilms, the NLRP3–IL-1β axis, macrophage polarization, RANKL/OPG imbalance, pyroptosis or necroptosis are biologically plausible, but most are not supported by robust randomized clinical trials in MRONJ [58,59,61–70,72–76,78,79]. Their timing, safety, dosage, route of delivery and long-term effects remain uncertain. In particular, excessive suppression of innate immune pathways may impair microbial clearance in lesions with active biofilm burden [58,59,68,78]. These strategies should therefore be regarded as mechanism-informed research directions rather than established clinical care.

Finally, the temporal relationships among the proposed pathological axes remain unclear. No uniform standard currently defines which mechanism dominates at a given disease phase, how the dominant axis changes over time or when cumulative pathological burden exceeds local reparative capacity. Future studies should combine longitudinal clinical follow-up, standardized sampling, multi-omics profiling, imaging analysis and functional validation to test the key nodes and feedback loops proposed in this framework [80–87]. Such work will be essential for determining whether the osteoimmuno-microbial model can improve risk stratification, early detection and phase-adapted management of MRONJ.

## Conclusions

Medication-related osteonecrosis of the jaw is unlikely to arise from a single pathogenic pathway. Rather, antiresorptive, antiangiogenic and other targeted therapies may create a vulnerable jawbone microenvironment in which reduced bone turnover, impaired vascular support, delayed mucosal repair and altered host defense lower the capacity for tissue recovery. Within this susceptible niche, microbial dysbiosis and biofilm persistence may provide continuous local stimulation, while inflammasome–IL-1β signaling, macrophage polarization, RANKL/OPG imbalance and inflammatory programmed cell death may reinforce one another.

The osteoimmuno-microbial feedback model proposed in this Review suggests that drugs may not act solely as direct triggers of osteonecrosis. Instead, they may reduce the buffering and reparative capacity of the local bone microenvironment, allowing infection- and inflammation-related stress to persist, amplify and become self-sustaining. Once microbial persistence, inflammatory dysregulation, bone remodeling imbalance and cell-death-associated tissue injury are interconnected, the lesion may progress from a potentially reversible local imbalance to persistent structural osteonecrosis.

From a translational perspective, two directions appear particularly promising. First, longitudinal risk stratification before clinical bone exposure may enable earlier identification of susceptible patients by integrating medication exposure, dental and systemic risk factors, microbial profiles, host immune markers and imaging features. Second, mechanism-informed local combination strategies that simultaneously control persistent biofilms while restoring inflammatory resolution and tissue repair may offer greater potential than antimicrobial therapy alone, particularly during intermediate disease phases before irreversible structural damage occurs. However, substantial barriers remain. Current MRONJ studies are frequently retrospective, cross-sectional, single-center and based on small cohorts, with heterogeneous sampling methods, disease definitions and treatment endpoints. Moreover, causal microbial and immune biomarkers have not yet been validated, and most targeted anti-biofilm, immunomodulatory and regenerative approaches remain preclinical or early translational. Future studies should therefore prioritize longitudinal multicenter validation, standardized biological and clinical endpoints, and phase-specific testing to determine whether these strategies can improve prevention, treatment response and recurrence control.

## Data Availability

No new datasets were generated or analyzed in this study. As this article is a narrative review based on previously published literature, data sharing is not applicable.

## References

[1] Marx RE . Pamidronate (Aredia) and zoledronate (Zometa) induced avascular necrosis of the jaws: a growing epidemic. J Oral Maxillofac Surg. 2003;61:1115–1117. doi: 10.1016/S0278-2391(03)00720-1 12966493

[2] Ruggiero SL , Dodson TB , Aghaloo T , et al. American association of oral and maxillofacial surgeons’ position paper on medication-related osteonecrosis of the Jaws-2022 update. J Oral Maxillofac Surg. 2022;80:920–943. doi: 10.1016/j.joms.2022.02.008 35300956

[3] Yarom N , Shapiro CL , Peterson DE , et al. Medication-related osteonecrosis of the jaw: MASCC/ISOO/ASCO clinical practice guideline. J Clin Oncol. 2019;37:2270–2290. doi: 10.1200/JCO.19.01186 31329513

[4] Khan AA , Morrison A , Hanley DA , et al. Diagnosis and management of osteonecrosis of the jaw: a systematic review and international consensus. J Bone Miner Res. 2015;30:3–23. doi: 10.1002/jbmr.2405 25414052

[5] He L , Sun X , Liu Z , et al. Pathogenesis and multidisciplinary management of medication-related osteonecrosis of the jaw. Int J Oral Sci. 2020;12:30. doi: 10.1038/s41368-020-00093-2 PMC7578793 33087699

[6] Anastasilakis AD , Pepe J , Napoli N , et al. Osteonecrosis of the jaw and antiresorptive agents in benign and malignant diseases: a critical review organized by the ECTS. J Clin Endocrinol Metab. 2022;107:1441–1460. doi: 10.1210/clinem/dgab888 PMC9016445 34922381

[7] Rodan GA , Fleisch HA . Bisphosphonates: mechanisms of action. J Clin Invest. 1996;97:2692–2696. doi: 10.1172/JCI118722 PMC507360 8675678

[8] Russell RGG . Bisphosphonates: the first 40 years. Bone. 2011;49:2–19. doi: 10.1016/j.bone.2011.04.022 21555003

[9] Coxon FP , Thompson K , Rogers MJ . Recent advances in understanding the mechanism of action of bisphosphonates. Curr Opin Pharmacol. 2006;6:307–312. doi: 10.1016/j.coph.2006.03.005 16650801

[10] Baron R , Ferrari S , Russell RGG . Denosumab and bisphosphonates: different mechanisms of action and effects. Bone. 2011;48:677–692. doi: 10.1016/j.bone.2010.11.020 21145999

[11] Boyle WJ , Simonet WS , Lacey DL . Osteoclast differentiation and activation. Nature. 2003;423:337–342. doi: 10.1038/nature01658 12748652

[12] Landesberg R , Woo V , Cremers S , et al. Potential pathophysiological mechanisms in osteonecrosis of the jaw. Ann NY Acad Sci. 2011;1218:62–79. doi: 10.1111/j.1749-6632.2010.05835.x PMC4477826 21291478

[13] Fantasia JE . The role of antiangiogenic therapy in the development of osteonecrosis of the jaw. Oral Maxillofac Surg Clin North Am. 2015;27:547–553. doi: 10.1016/j.coms.2015.06.004 26515736

[14] Saad F , Brown J , Van Poznak C , et al. Incidence, risk factors, and outcomes of osteonecrosis of the jaw: integrated analysis from three blinded active-controlled phase III trials in cancer patients with bone metastases. Ann Oncol. 2012;23:1341–1347. doi: 10.1093/annonc/mdr435 21986094

[15] Dodson TB . The frequency of medication-related osteonecrosis of the jaw and its associated risk factors. Oral Maxillofac Surg Clin North Am. 2015;27:509–516. doi: 10.1016/j.coms.2015.06.003 26362367

[16] McGowan K , McGowan T , Ivanovski S . Risk factors for medication-related osteonecrosis of the jaws: a systematic review. Oral Dis. 2018;24:527–536. doi: 10.1111/odi.12708 28656643

[17] Soutome S , Otsuru M , Hayashida S , et al. Relationship between tooth extraction and development of medication-related osteonecrosis of the jaw in cancer patients. Sci Rep. 2021;11:17226. doi: 10.1038/s41598-021-96480-8 PMC8390686 34446755

[18] Hasegawa T , Hayashida S , Kondo E , et al. Medication-related osteonecrosis of the jaw after tooth extraction in cancer patients: a multicenter retrospective study. Osteoporos Int. 2019;30:231–239. doi: 10.1007/s00198-018-4746-8 30406309

[19] Otto S , Tröltzsch M , Jambrovic V , Panya S , Probst F , Ristow O , Ehrenfeld M , Pautke C . Tooth extraction in patients receiving oral or intravenous bisphosphonate administration: a trigger for BRONJ development? J Cranio Maxillof Surg. 2015;43(6):847–854. doi: 10.1016/j.jcms.2015.03.039 25958767

[20] Tsao C , Darby I , Ebeling PR , et al. Oral health risk factors for bisphosphonate-associated jaw osteonecrosis. J Oral Maxillof Surg. 2013;71:1360–1366. doi: 10.1016/j.joms.2013.02.016 23582590

[21] Katsarelis H , Shah NP , Dhariwal DK , et al. Infection and medication-related osteonecrosis of the jaw. J Dent Res. 2015;94:534–539. doi: 10.1177/0022034515572021 25710950

[22] Otto S , Aljohani S , Fliefel R , et al. Infection as an important factor in medication-related osteonecrosis of the jaw (MRONJ). Medicina. 2021;57:463. doi: 10.3390/medicina57050463 PMC8151678 34065104

[23] Hansen T , Kunkel M , Weber A , et al. Osteonecrosis of the jaws in patients treated with bisphosphonates – histomorphologic analysis in comparison with infected osteoradionecrosis. J Oral Pathol Med. 2006;35:155–160. doi: 10.1111/j.1600-0714.2006.00391.x 16454811

[24] Sedghizadeh PP , Kumar SK , Gorur A , et al. Microbial biofilms in osteomyelitis of the jaw and osteonecrosis of the jaw secondary to bisphosphonate therapy. JADA. 2009;140:1259–1265. doi: 10.14219/jada.archive.2009.0049 19797556

[25] Sedghizadeh PP , Kumar SK , Gorur A , et al. Identification of microbial biofilms in osteonecrosis of the jaws secondary to bisphosphonate therapy. J Oral Maxillof Surg. 2008;66:767–775. doi: 10.1016/j.joms.2007.11.035 18355603

[26] Russmueller G , Seemann R , Weiss K , et al. The association of medication-related osteonecrosis of the jaw with actinomyces spp. Infection. Sci Rep. 2016;6:31604. doi: 10.1038/srep31604 PMC4987681 27530150

[27] Brody A , Scheich B , Dobo-Nagy C . Targeted histological evaluation shows high incidence of actinomyces infection in medication-related osteonecrosis of the jaws. Sci Rep. 2022;12:3406. doi: 10.1038/s41598-022-07375-1 PMC8888741 35233034

[28] Pushalkar S , Li X , Kurago Z , et al. Oral microbiota and host innate immune response in bisphosphonate-related osteonecrosis of the jaw. Int J Oral Sci. 2014;6:219–226. doi: 10.1038/ijos.2014.46 PMC5153588 25105817

[29] Hallmer F , Bjørnland T , Andersson G , et al. Bacterial diversity in medication-related osteonecrosis of the jaw. Oral Surg Oral Med Oral Pathol Oral Radiol. 2017;123:436–444. doi: 10.1016/j.oooo.2016.11.011 28159588

[30] Wei X , Pushalkar S , Estilo C , et al. Molecular profiling of oral microbiota in jawbone samples of bisphosphonate-related osteonecrosis of the jaw. Oral Dis. 2012;18:602–612. doi: 10.1111/j.1601-0825.2012.01916.x PMC7167636 22443347

[31] Kim H-Y , Jung Y-S , Park W , et al. Can medication-related osteonecrosis of the jaw be attributed to specific microorganisms through oral microbiota analyses? A preliminary study. BMC Oral Health. 2024;24:160. doi: 10.1186/s12903-024-03945-z PMC10832156 38302952

[32] Ewald F , Wuesthoff F , Koehnke R , et al. Retrospective analysis of bacterial colonization of necrotic bone and antibiotic resistance in 98 patients with medication-related osteonecrosis of the jaw (MRONJ). Clin Oral Invest. 2021;25:2801–2809. doi: 10.1007/s00784-020-03595-9 PMC8060223 33006027

[33] Tetradis S , Allen MR , Ruggiero SL . Pathophysiology of medication-related osteonecrosis of the Jaw—A minireview. JBMR Plus. 2023;7:e10785. doi: 10.1002/jbm4.10785 PMC10443081 37614299

[34] Zhang W , Gao L , Ren W , et al. The role of the immune response in the development of medication-related osteonecrosis of the jaw. Front Immunol. 2021;12:606043. doi: 10.3389/fimmu.2021.606043 PMC7947359 33717086

[35] Roato I , Mauceri R , Notaro V , et al. Immune dysfunction in medication-related osteonecrosis of the jaw. IJMS. 2023;24:7948. doi: 10.3390/ijms24097948 PMC10177780 37175652

[36] Patntirapong S , Poolgesorn M . Alteration of macrophage viability, differentiation, and function by bisphosphonates. Oral Dis. 2018;24:1294–1302. doi: 10.1111/odi.12908 29869362

[37] Hagelauer N , Pabst AM , Ziebart T , et al. In vitro effects of bisphosphonates on chemotaxis, phagocytosis, and oxidative burst of neutrophil granulocytes. Clin Oral Invest. 2015;19:139–148. doi: 10.1007/s00784-014-1219-0 24668343

[38] Zhu W , Xu R , Du J , et al. Zoledronic acid promotes TLR‐4‐mediated M1 macrophage polarization in bisphosphonate‐related osteonecrosis of the jaw. FASEB J. 2019;33:5208–5219. doi: 10.1096/fj.201801791RR 30624969

[39] Lu Y-C , Yeh W-C , Ohashi PS . LPS/TLR4 signal transduction pathway. Cytokine. 2008;42:145–151. doi: 10.1016/j.cyto.2008.01.006 18304834

[40] Swanson KV , Deng M , Ting JP-Y . The NLRP3 inflammasome: molecular activation and regulation to therapeutics. Nat Rev Immunol. 2019;19:477–489. doi: 10.1038/s41577-019-0165-0 PMC7807242 31036962

[41] Zhang Q , Yu W , Lee S , et al. Bisphosphonate induces osteonecrosis of the jaw in diabetic mice via NLRP3/Caspase-1-Dependent IL-1β mechanism. J Bone Miner Res. 2015;30:2300–2312. doi: 10.1002/jbmr.2577 PMC4941639 26081624

[42] Dewhirst FE , Chen T , Izard J , et al. The human oral microbiome. J Bacteriol. 2010;192:5002–5017. doi: 10.1128/JB.00542-10 PMC2944498 20656903

[43] Baker JL , Mark Welch JL , Kauffman KM , et al. The oral microbiome: diversity, biogeography and human health. Nat Rev Microbiol. 2024;22:89–104. doi: 10.1038/s41579-023-00963-6 PMC11084736 37700024

[44] Lamont RJ , Koo H , Hajishengallis G . The oral microbiota: dynamic communities and host interactions. Nat Rev Microbiol. 2018;16:745–759. doi: 10.1038/s41579-018-0089-x PMC6278837 30301974

[45] Wade WG . The oral microbiome in health and disease. Pharmacol Res. 2013;69:137–143. doi: 10.1016/j.phrs.2012.11.006 23201354

[46] Du W , Yang M , Kim T , et al. Indigenous microbiota protects development of medication-related osteonecrosis induced by periapical disease in mice. Int J Oral Sci. 2022;14:16. doi: 10.1038/s41368-022-00166-4 PMC8934872 35307731

[47] Jiang A , Zhang Z , Qiu X , et al. Medication-related osteonecrosis of the jaw (MRONJ): a review of pathogenesis hypothesis and therapy strategies. Arch Toxicol. 2024;98:689–708. doi: 10.1007/s00204-023-03653-7 38155341

[48] Kobayashi Y , Hiraga T , Ueda A , et al. Zoledronic acid delays wound healing of the tooth extraction socket, inhibits oral epithelial cell migration, and promotes proliferation and adhesion to hydroxyapatite of oral bacteria, without causing osteonecrosis of the jaw, in mice. J Bone Miner Metab. 2010;28:165–175. doi: 10.1007/s00774-009-0128-9 19882100

[49] Scheper MA , Badros A , Chaisuparat R , et al. Effect of zoledronic acid on oral fibroblasts and epithelial cells: a potential mechanism of bisphosphonate-associated osteonecrosis. Br J Haematol. 2009;144:667–676. doi: 10.1111/j.1365-2141.2008.07504.x PMC2739302 19036117

[50] Ohnuki H , Izumi K , Terada M , et al. Zoledronic acid induces S-phase arrest via a DNA damage response in normal human oral keratinocytes. Arch Oral Biol. 2012;57:906–917. doi: 10.1016/j.archoralbio.2011.11.015 22172403

[51] Ravosa MJ , Ning J , Liu Y , et al. Bisphosphonate effects on the behaviour of oral epithelial cells and oral fibroblasts. Arch Oral Biol. 2011;56:491–498. doi: 10.1016/j.archoralbio.2010.11.003 21146154

[52] Badros AZ , Meddeb M , Weikel D , et al. Prospective observational study of bisphosphonate-related osteonecrosis of the jaw in multiple myeloma: microbiota profiling and cytokine expression. Front Oncol. 2021;11:704722. doi: 10.3389/fonc.2021.704722 PMC8263936 34249765

[53] Hajishengallis G . Periodontitis: from microbial immune subversion to systemic inflammation. Nat Rev Immunol. 2015;15:30–44. doi: 10.1038/nri3785 PMC4276050 25534621

[54] Brennan CA , Garrett WS . Fusobacterium nucleatum — symbiont, opportunist and oncobacterium. Nat Rev Microbiol. 2019;17:156–166. doi: 10.1038/s41579-018-0129-6 PMC6589823 30546113

[55] Kolenbrander PE , Palmer RJ , Rickard AH , et al. Bacterial interactions and successions during plaque development. Periodontol 2000. 2006;42:47–79. doi: 10.1111/j.1600-0757.2006.00187.x 16930306

[56] Xu W , Zhou W , Wang H , et al. Roles of porphyromonas gingivalis and its virulence factors in periodontitis. Adv Protein Chem Struct Biol. 2020;120:45–84. doi: 10.1016/bs.apcsb.2019.12.001 PMC8204362 32085888

[57] Yahara H , Hiraki A , Maruoka Y , et al. Shotgun metagenome sequencing identification of a set of genes encoded by actinomyces associated with medication-related osteonecrosis of the jaw. PLoS One. 2020;15:e0241676. doi: 10.1371/journal.pone.0241676 PMC7703938 33253207

[58] Flemming H-C , Wingender J , Szewzyk U , et al. Biofilms: an emergent form of bacterial life. Nat Rev Microbiol. 2016;14:563–575. doi: 10.1038/nrmicro.2016.94 27510863

[59] Liu HY , Prentice EL , Webber MA . Mechanisms of antimicrobial resistance in biofilms. npj Antimicrob Resist. 2024;2:27. doi: 10.1038/s44259-024-00046-3 PMC11445061 39364333

[60] Hall-Stoodley L , Costerton JW , Stoodley P . Bacterial biofilms: from the natural environment to infectious diseases. Nat Rev Microbiol. 2004;2:95–108. doi: 10.1038/nrmicro821 15040259

[61] Gkouveris I , Soundia A , Gouveris P , Zouki D , Hadaya D , Tetradis S . Macrophage involvement in medication-related osteonecrosis of the jaw (MRONJ): a comprehensive, short review. Cancers. 2022;14(2):330. doi: 10.3390/cancers14020330 PMC8773732 35053492

[62] Paschalidi P , Gkouveris I , Soundia A , et al. The role of M1 and M2 macrophage polarization in progression of medication-related osteonecrosis of the jaw. Clin Oral Invest. 2021;25:2845–2857. doi: 10.1007/s00784-020-03602-z PMC7982347 32964311

[63] Soundia A , Elzakra N , Hadaya D , et al. Macrophage polarization during MRONJ development in mice. J Dent Res. 2024;103:899–907. doi: 10.1177/00220345241258990 PMC11465330 39104155

[64] Okamoto K , Nakashima T , Shinohara M , et al. Osteoimmunology: the conceptual framework unifying the immune and skeletal systems. Physiol Rev. 2017;97:1295–1349. doi: 10.1152/physrev.00036.2016 28814613

[65] Takegahara N , Kim H , Choi Y . RANKL biology. Bone. 2022;159:116353. doi: 10.1016/j.bone.2022.116353 PMC9035122 35181574

[66] Amarasekara DS , Yun H , Kim S , et al. Regulation of osteoclast differentiation by cytokine networks. Immune Netw. 2018;18:e8. doi: 10.4110/in.2018.18.e8 PMC5833125 29503739

[67] Takayanagi H , Kim S , Koga T , et al. Induction and activation of the transcription factor NFATc1 (NFAT2) integrate RANKL signaling in terminal differentiation of osteoclasts. Dev Cell. 2002;3:889–901. doi: 10.1016/S1534-5807(02)00369-6 12479813

[68] Zheng D , Liwinski T , Elinav E . Inflammasome activation and regulation: toward a better understanding of complex mechanisms. Cell Discov. 2020;6:36. doi: 10.1038/s41421-020-0167-x PMC7280307 32550001

[69] Shi J , Zhao Y , Wang K , et al. Cleavage of GSDMD by inflammatory caspases determines pyroptotic cell death. Natur. 2015;526:660–665. doi: 10.1038/nature15514 26375003

[70] He W , Wan H , Hu L , et al. Gasdermin D is an executor of pyroptosis and required for interleukin-1β secretion. Cell Res. 2015;25:1285–1298. doi: 10.1038/cr.2015.139 PMC4670995 26611636

[71] Li L , Huang H , He Y , et al. Cell-free DNA in sepsis: from molecular insights to clinical management. Mil Med Res. 2025;12:85. doi: 10.1186/s40779-025-00668-2 PMC12670778 41327452

[72] Dinarello CA . Overview of the IL ‐1 family in innate inflammation and acquired immunity. Immunol Rev. 2018;281:8–27. 10.1111/imr.12621 PMC5756628 29247995

[73] Pasparakis M , Vandenabeele P . Necroptosis and its role in inflammation. Nature. 2015;517:311–320. doi: 10.1038/nature14191 25592536

[74] Galluzzi L , Kepp O , Chan FK-M , et al. Necroptosis: mechanisms and relevance to disease. Annu Rev Pathol. 2017;12:103–130. doi: 10.1146/annurev-pathol-052016-100247 PMC5786374 27959630

[75] Kim J-W , Kong S , Kwak MK , et al. Medication-related osteonecrosis of the jaw: an evidence-based 2025 position statement from a Korean multidisciplinary task force. JKAOMS. 2025;51:333–353. doi: 10.5125/jkaoms.2025.51.6.333 PMC12766015 41466542

[76] Chen D , Li D , Su L , et al. Stimuli-responsive, antimicrobial-loaded nanocarriers for oral biofilm control and microbiome restoration. Int J Oral Sci. 2026;18:17. doi: 10.1038/s41368-025-00422-3 PMC12891596 41667443

[77] Feng J , Liu Y , Gong L , et al. Targeting intratumoral bacteria reverses MHC-I suppression and enhances nano-based chemoimmunotherapy in colorectal cancer. Mil Med Res. 2026;13:100036. doi: 10.1016/j.mmr.2026.100036 PMC13185994 42164099

[78] Mangan MSJ , Olhava EJ , Roush WR , et al. Targeting the NLRP3 inflammasome in inflammatory diseases. Nat Rev Drug Discov. 2018;17:588–606. doi: 10.1038/nrd.2018.97 30026524

[79] Mifflin L , Ofengeim D , Yuan J . Receptor-interacting protein kinase 1 (RIPK1) as a therapeutic target. Nat Rev Drug Discov. 2020;19:553–571. doi: 10.1038/s41573-020-0071-y PMC7362612 32669658

[80] Lorenzo-Pouso AI , Lorenzo‐Pouso AI , Bravo SB , et al. Quantitative proteomics in medication-related osteonecrosis of the jaw: a proof-of-concept study. Oral Dis. 2023;29:2117–2129. doi: 10.1111/odi.14201 35377498

[81] Schwartzová V , Laputková G , Talian I , et al. Insights into medication-induced osteonecrosis of the jaw through the application of salivary proteomics and bioinformatics. IJMS. 2024;25:12405. doi: 10.3390/ijms252212405 PMC11594355 39596473

[82] Laputková G , Talian I , Schwartzová V . Medication-related osteonecrosis of the jaw: a systematic review and a bioinformatic analysis. IJMS. 2023;24:16745. doi: 10.3390/ijms242316745 PMC10706386 38069068

[83] Bojtor B , Balla B , Vaszilko M , et al. Genetic background of medication-related osteonecrosis of the jaw: current evidence and future perspectives. IJMS. 2024;25:10488. doi: 10.3390/ijms251910488 PMC11477157 39408816

[84] Mine Y , Okazaki S , Yamasaki S , et al. Artificial intelligence for medication-related osteonecrosis of the jaw: a scoping review. Oral Surg Oral Med Oral Pathol Oral Radiol. 2025;140:405–415. doi: 10.1016/j.oooo.2025.03.004 40393880

[85] Kwack DW , Park SM . Prediction of medication-related osteonecrosis of the jaw (MRONJ) using automated machine learning in patients with osteoporosis associated with dental extraction and implantation: a retrospective study. JKAOMS. 2023;49:135–141. doi: 10.5125/jkaoms.2023.49.3.135 PMC10318313 37394932

[86] Toriumi S , Shimokawa K , Yamamoto M , et al. Development of a medication-related osteonecrosis of the jaw prediction model using the FDA adverse event reporting system database and machine learning. Pharmaceuticals. 2025;18:423. doi: 10.3390/ph18030423 PMC11945420 40143199

[87] Konishi M , Nishi H , Kawaguchi H , et al. Radiomics-based classification of medication-related osteonecrosis of the jaw using panoramic radiographs. Oral Radiol. 2025;41:507–516. doi: 10.1007/s11282-025-00826-1 PMC12423229 40323521

[88] Woo S-B , Hellstein JW , Kalmar JR . Systematic review: bisphosphonates and osteonecrosis of the jaws. Ann Intern Med. 2006;144:753–761. doi: 10.7326/0003-4819-144-10-200605160-00009 16702591

[89] Khosla S , Burr D , Cauley J , et al. Bisphosphonate-associated osteonecrosis of the jaw: report of a task force of the American society for bone and mineral research. J Bone Miner Res. 2007;22:1479–1491. doi: 10.1359/jbmr.0707onj 17663640

[90] Hoff AO , Toth BB , Altundag K , et al. Frequency and risk factors associated with osteonecrosis of the jaw in cancer patients treated with intravenous bisphosphonates. J Bone Miner Res. 2008;23:826–836. doi: 10.1359/jbmr.080205 PMC2677083 18558816

[91] Vahtsevanos K , Kyrgidis A , Verrou E , et al. Longitudinal cohort study of risk factors in cancer patients of bisphosphonate-related osteonecrosis of the jaw. JCO. 2009;27:5356–5362. doi: 10.1200/JCO.2009.21.9584 19805682

[92] Barasch A , Cunha-Cruz J , Curro F , et al. Risk factors for osteonecrosis of the jaws: a case-control study from the CONDOR dental PBRN. J Dent Res. 2011;90:439–444. doi: 10.1177/0022034510397196 PMC3144129 21317246

[93] Lo JC , O'Ryan FS , Gordon NP , et al. Prevalence of osteonecrosis of the jaw in patients with oral bisphosphonate exposure. J Oral Maxillof Surg. 2010;68:243–253. doi: 10.1016/j.joms.2009.03.050 PMC10159647 19772941

[94] Fliefel R , Tröltzsch M , Kühnisch J , et al. Treatment strategies and outcomes of bisphosphonate-related osteonecrosis of the jaw (BRONJ) with characterization of patients: a systematic review. Int J Oral Maxillofac Surg. 2015;44:568–585. doi: 10.1016/j.ijom.2015.01.026 25726090

[95] Beth-Tasdogan NH , Mayer B , Hussein H , et al. Interventions for managing medication-related osteonecrosis of the jaw. Cochrane Database Syst Rev. 2022;2022(7). doi: 10.1002/14651858.CD012432.pub3 PMC9309005 35866376

[96] Bi Y , Gao Y , Ehirchiou D , et al. Bisphosphonates cause osteonecrosis of the jaw-like disease in mice. Am J Pathol. 2010;177:280–290. doi: 10.2353/ajpath.2010.090592 PMC2893671 20472893

[97] Aghaloo TL , Cheong S , Bezouglaia O , et al. RANKL inhibitors induce osteonecrosis of the jaw in mice with periapical disease. J Bone Miner Res. 2014;29:843–854. doi: 10.1002/jbmr.2097 PMC4476544 24115073

[98] Williams DW , Lee C , Kim T , et al. Impaired bone resorption and woven bone formation are associated with development of osteonecrosis of the jaw-like lesions by bisphosphonate and Anti–Receptor activator of NF-κB ligand antibody in mice. Am J Pathol. 2014;184:3084–3093. doi: 10.1016/j.ajpath.2014.07.010 PMC4215026 25173134

[99] Burr D , Allen M . Mandibular necrosis in beagle dogs treated with bisphosphonates. Orthod Craniofacial Res. 2009;12:221–228. doi: 10.1111/j.1601-6343.2009.01456.x PMC2765865 19627524

[100] Socransky SS , Haffajee AD , Cugini MA , et al. Microbial complexes in subgingival plaque. J Clin Periodontol. 1998;25:134–144. doi: 10.1111/j.1600-051X.1998.tb02419.x 9495612

[101] Marsh PD . Dental plaque as a biofilm and a microbial community – implications for health and disease. BMC Oral Health. 2006;6:S14. doi: 10.1186/1472-6831-6-S1-S14 PMC2147593 16934115

[102] Jakubovics NS , Goodman SD , Mashburn-Warren L , et al. The dental plaque biofilm matrix. Periodontol 2000. 2021;86:32–56. doi: 10.1111/prd.12361 PMC9413593 33690911

[103] Koo H , Allan RN , Howlin RP , et al. Targeting microbial biofilms: current and prospective therapeutic strategies. Nat Rev Microbiol. 2017;15:740–755. doi: 10.1038/nrmicro.2017.99 PMC5685531 28944770

[104] Takayanagi H . Osteoimmunology: shared mechanisms and crosstalk between the immune and bone systems. Nat Rev Immunol. 2007;7:292–304. doi: 10.1038/nri2062 17380158

[105] Walsh MC , Takegahara N , Kim H , et al. Updating osteoimmunology: regulation of bone cells by innate and adaptive immunity. Nat Rev Rheumatol. 2018;14:146–156. doi: 10.1038/nrrheum.2017.213 PMC5821527 29323344

[106] Lacey DL , Timms E , Tan H , et al. Osteoprotegerin ligand is a cytokine that regulates osteoclast differentiation and activation. Cell. 1998;93:165–176. doi: 10.1016/S0092-8674(00)81569-X 9568710

[107] Yasuda H , Shima N , Nakagawa N , et al. Osteoclast differentiation factor is a ligand for osteoprotegerin/osteoclastogenesis-inhibitory factor and is identical to TRANCE/RANKL. Proc Natl Acad Sci USA. 1998;95:3597–3602. doi: 10.1073/pnas.95.7.3597 PMC19881 9520411

[108] Schroder K , Tschopp J . The inflammasomes. Cell. 2010;140:821–832. doi: 10.1016/j.cell.2010.01.040 20303873

[109] Broz P , Dixit VM . Inflammasomes: mechanism of assembly, regulation and signalling. Nat Rev Immunol. 2016;16:407–420. doi: 10.1038/nri.2016.58 27291964

[110] Galluzzi L , Vitale I , Aaronson SA , et al. Molecular mechanisms of cell death: recommendations of the nomenclature committee on cell death 2018. Cell Death Differ. 2018;25:486–541. doi: 10.1038/s41418-017-0012-4 PMC5864239 29362479

[111] Berghe TV , Linkermann A , Jouan-Lanhouet S , et al. Regulated necrosis: the expanding network of non-apoptotic cell death pathways. Nat Rev Mol Cell Biol. 2014;15:135–147. doi: 10.1038/nrm3737 24452471

[112] Gillies RJ , Kinahan PE , Hricak H . Radiomics: images are more than pictures, they are data. Radiology. 2016;278:563–577. doi: 10.1148/radiol.2015151169 PMC4734157 26579733

[113] Lambin P , Leijenaar RT , Deist TM , et al. Radiomics: the bridge between medical imaging and personalized Medicine. Nat Rev Clin Oncol. 2017;14:749–762. doi: 10.1038/nrclinonc.2017.141 28975929

[114] Topol EJ . High-performance Medicine: the convergence of human and artificial intelligence. Nat Med. 2019;25:44–56. doi: 10.1038/s41591-018-0300-7 30617339

